# Conventional and frugal methods of estimating COVID-19-related excess deaths and undercount factors

**DOI:** 10.1038/s41598-024-57634-6

**Published:** 2024-05-06

**Authors:** Abhishek M. Dedhe, Aakash A. Chowkase, Niramay V. Gogate, Manas M. Kshirsagar, Rohan Naphade, Atharv Naphade, Pranav Kulkarni, Mrunmayi Naik, Aarya Dharm, Soham Raste, Shravan Patankar, Chinmay M. Jogdeo, Aalok Sathe, Soham Kulkarni, Vibha Bapat, Rohinee Joshi, Kshitij Deshmukh, Subhash Lele, Kody J. Manke-Miller, Jessica F. Cantlon, Pranav S. Pandit

**Affiliations:** 1 JPF Analytics, Jnana Prabodhini Foundation, Murrieta, CA USA; 2 Department of Psychology, Carnegie Mellon University, Pittsburgh, PA USA; 3 Center for the Neural Basis of Cognition, Carnegie Mellon University, Pittsburgh, PA USA; 4 Department of Psychology, University of California, Berkeley, CA USA; 5 Department of Physics and Astronomy, Texas Tech University, Lubbock, TX USA; 6 Institute of Clinical Neurobiology, University Hospital Würzburg, Würzburg, Germany; 7 Department of Electrical Engineering, California Institute of Technology, Pasadena, CA USA; 8 School of Computer Science and Engineering, University of Washington, Seattle, WA USA; 9 Department of Mathematics, University of Illinois, Chicago, IL USA; 10 College of Pharmacy, University of Nebraska Medical Center, Omaha, NE USA; 11 Department of Brain and Cognitive Sciences, Massachusetts Institute of Technology, Cambridge, MA USA; 12 Troy High School, Fullerton, CA USA; 13 Department of Biology, Indian Institute of Science Education and Research, Pune, Maharashtra India; 14 Department of Mathematics, Indian Institute of Technology Bombay, Mumbai, Maharashtra India; 15 Division of Molecular and Cellular Function, School of Biological Sciences, University of Manchester, Manchester, Greater Manchester UK; 16 Department of Molecular Physiology and Biophysics, Pappajohn Biomedical Discovery Building (PBDB), University of Iowa, Iowa City, IA USA; 17 Department of Mathematical and Statistical Sciences, University of Alberta, Edmonton, AB Canada; 18 Department of Population Health and Reproduction, School of Veterinary Medicine, University of California, Davis, Davis, CA USA

**Keywords:** Statistical modeling, Wisdom of crowds, COVID-19, Excess mortality estimation, Frugal science, Psychology, Diseases

## Abstract

Across the world, the officially reported number of COVID-19 deaths is likely an undercount. Establishing true mortality is key to improving data transparency and strengthening public health systems to tackle future disease outbreaks. In this study, we estimated excess deaths during the COVID-19 pandemic in the Pune region of India. Excess deaths are defined as the number of additional deaths relative to those expected from pre-COVID-19-pandemic trends. We integrated data from: (a) epidemiological modeling using pre-pandemic all-cause mortality data, (b) discrepancies between media-reported death compensation claims and official reported mortality, and (c) the “wisdom of crowds” public surveying. Our results point to an estimated 14,770 excess deaths [95% CI 9820–22,790] in Pune from March 2020 to December 2021, of which 9093 were officially counted as COVID-19 deaths. We further calculated the undercount factor—the ratio of excess deaths to officially reported COVID-19 deaths. Our results point to an estimated undercount factor of 1.6 [95% CI 1.1–2.5]. Besides providing similar conclusions about excess deaths estimates across different methods, our study demonstrates the utility of frugal methods such as the analysis of death compensation claims and the wisdom of crowds in estimating excess mortality.

## Introduction

Accurate and trustworthy data are critical for understanding, mitigating, and preventing complex problems. During disease outbreaks, precise mortality data are essential for facilitating optimal resource allocation, conducting retrospective evaluations of disease mitigation measures, and effectively planning for—and perhaps potentially preventing—future epidemics and other public health emergencies^1,2^. However, there are widespread concerns about the lack of reliable mortality estimates during large-scale disease outbreaks, and this concern was prevalent during the COVID-19 pandemic^3–6^. Skepticism about the validity of official COVID-19 mortality data arises from a lack of rigorous testing, absence of medical certification, deaths occurring outside formal healthcare systems, and other indirect pandemic-related deaths that occurred due to delays or lack of access to healthcare, reduced hospital capacity, increased risk of other diseases, and post-COVID-19 complications^3,4,7,8^. In response, national and international health authorities, epidemiologists, and journalists across the world have estimated the number of additional deaths during the COVID-19 pandemic relative to those expected based on trends from pre-pandemic times, also known as *excess mortality*^4,5,7,9–14^. Excess mortality estimates are conventionally computed using a range of statistical and epidemiological models^4,5,7,8,10–25^. However, these models rely on plentiful, high-quality, and timely pre-pandemic mortality data. Such data are particularly difficult to obtain in resource-constrained settings where robust and resilient data infrastructures are limited^2,3,26–28^. In such circumstances, researchers have estimated COVID-19-related excess mortality using innovative alternatives such as post-mortem PCR tests, large household surveys, satellite imagery of cemeteries, obituary notifications, funeral data, cremation counts, burial numbers, death insurance claims, verbal autopsies, and investigative journalism^10,29–36^. Although useful, many of these alternative methods remain resource-intensive, and thus prompt a need for frugal fact-finding approaches in addition to traditional data-collection techniques.

To that end, the *wisdom of crowds* approach may potentially be useful in estimating excess mortality. The wisdom of crowds refers to the counterintuitive accuracy of aggregated cognitive estimates. Cognitive estimation is the human ability to provide reasonable answers to questions for which specific answers are not readily available^37–39,126^. Many everyday human behaviors depend on successful cognitive estimation (e.g., planning out how many clothes to pack for a trip). Such everyday cognitive estimation scenarios tap into a range of psychological processes such as reasoning, working memory, cognitive flexibility, mental imagery, and problem-solving^40–45^. Cognitive estimation has therefore been applied across clinical populations to assess patients with brain lesions and other psychiatric conditions^46–56^. Such neuropsychological studies have made progress in describing the neural underpinnings of cognitive estimation. Parallel research efforts have investigated cognitive estimation in the context of education and neurocognitive developmental disorders^57–61^. Such research has shown how the accuracy of cognitive estimation is sensitive to experiential factors including socioeconomic status, reading habits, quality of education, and media exposure^62^. Even though individual human judgment and decision-making are often biased and susceptible to influence from a range of cognitive, emotional, socioeconomic, and political factors^27,63–66^, a growing body of research points to the wisdom of crowds. This frugal method has been widely used across multiple domains including neuropsychology, business, finance, economics, election polling, public policy, and global geopolitics^37,39,59,67–77^. Specifically, in the context of epidemiology, public health surveillance, and the COVID-19 pandemic, this method has been used to predict future outbreaks, vaccination uptake, disease caseload, infection hotspots, and overall disease severity^72,78–86^. Despite the potential of cognitive estimation, the utility of this method has not been widely tested to estimate pandemic-associated excess mortality, a gap this study aims to fill.

In this study, we investigated whether COVID-19-related excess mortality estimates using multiple methods were similar to each other. We focused on three conventional statistical and epidemiological models: (a) a simple averaging technique^18,87^, (b) the Farrington surveillance algorithm^20^, and (c) an overdispersed Poisson model^88^, along with two novel frugal methods to estimate COVID-19-related excess mortality: (d) analyzing media reports about discrepancies between official mortality data and death compensation claims, and (e) the wisdom of crowds public surveying. Similar estimates obtained from different methods would establish the use of frugal methods in estimating pandemic-related excess mortality and other unknown public health-related statistics, especially in resource-constrained settings.

**Figure 1:**
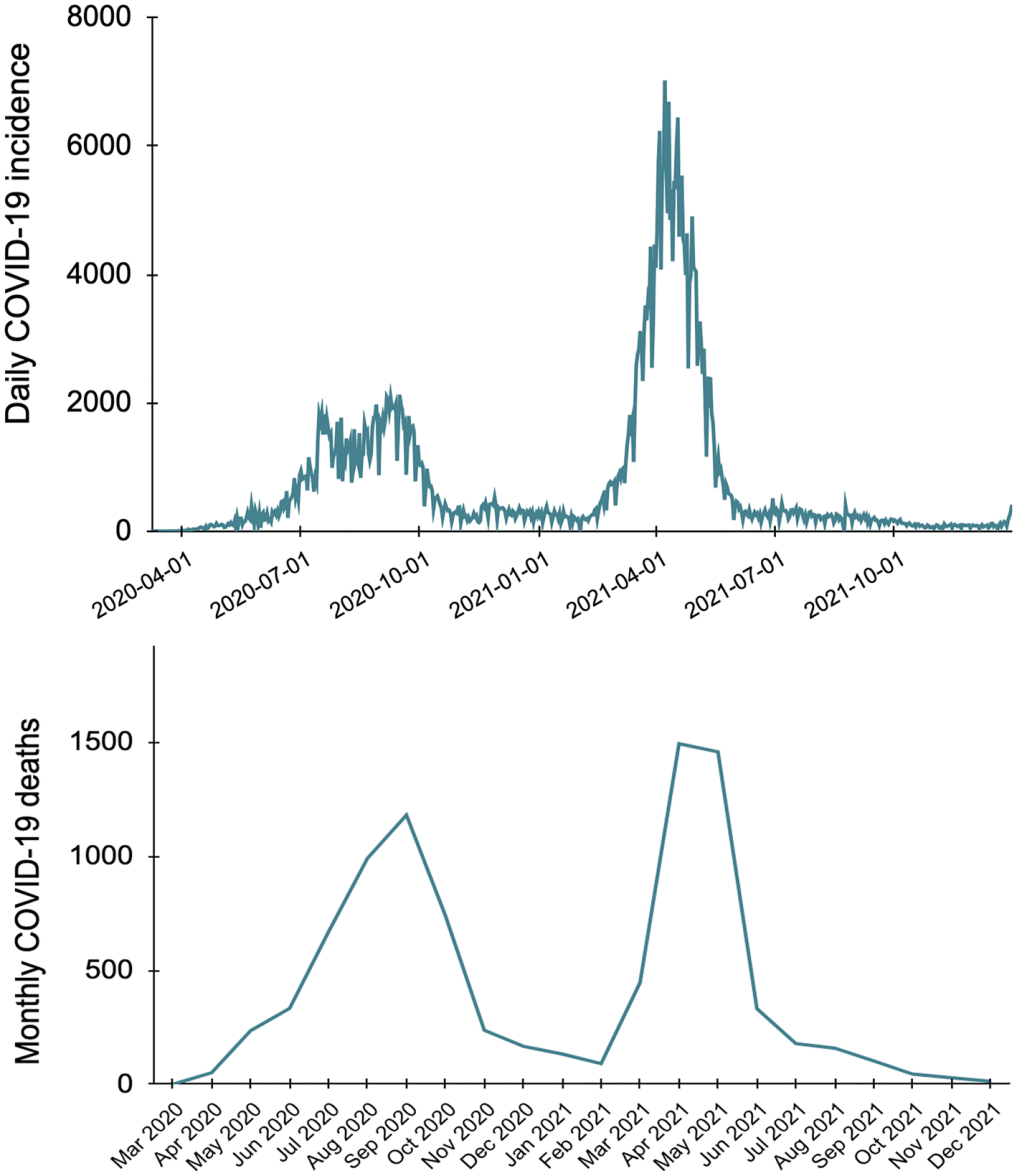
Officially reported COVID-19 incidence and deaths in Pune. Top: Daily COVID-19 incidence in Pune from March 2020 through December 2021. Bottom: Monthly COVID-19 deaths reported in Pune from March 2020 through December 2021.

In our case study, we focused on the Pune Municipal Corporation region (henceforth simply referred to as ‘Pune’) in the state of Maharashtra, India. Pune is the eighth-largest metropolitan area of India with a population of 5 million people^89^. Of them, around 40% of inhabitants (~ 2 million) reside in urban slums^90^, with an additional floating population of migrants from surrounding rural areas. Between 1st January 2020 and 31st December 2021, Pune officially reported more than half a million COVID-19 cases and 9093 COVID-19 deaths over two successive waves (Fig. 1, Fig. S1)^91^. Despite a large number of officially reported cases and deaths, Pune is one of the few large Indian cities for which pandemic-associated excess mortality has not been determined. We, therefore, estimated COVID-19-related excess deaths in Pune. Our results point to an estimated 14,770 excess deaths [95% CI: 9820–22,790] in Pune from March 2020 to December 2021, of which 9093 were officially counted as COVID-19 deaths. This translates to an estimated undercount factor—the ratio of estimated excess deaths to officially reported COVID-19 deaths—of 1.6 [95% CI: 1.1–2.5] for Pune from March 2020 to December 2021. In other words, we estimate that Pune experienced 60% more COVID-19-related deaths than officially reported. We found that excess death estimates from diverse methods—both conventional and frugal—were within the margins of error of each other. Thus, our study provides evidence about excess death estimates from diverse approaches and demonstrates the utility of non-traditional frugal methods such as the analysis of media-reported death compensation claims and the wisdom of crowds in estimating excess mortality. Our study also reinforces the potential of collective cognitive estimation as an untapped theoretical avenue for computational social science, neuroscience, cognitive and behavioral science, and other life sciences. Finally, our study highlights the practical importance of the wisdom of crowds and other frugal estimation methods in generating equitable solutions to credible fact-finding, especially in resource-constrained settings where robust data infrastructures are unaffordable.

## Methods

We adopted a multi-method approach to estimate COVID-19-related excess deaths in Pune from March 2020 to December 2021 by combining estimates from three methods: (a) statistical and epidemiological modeling with pre-pandemic mortality data, (b) analyzing media reports about discrepancies between official mortality data and death compensation claims, and (c) wisdom of crowds public surveying. Within statistical and epidemiological methods, we used three models: (a) a simple averaging technique^18,87^, (b) the Farrington surveillance algorithm^20^, and (c) an overdispersed Poisson model^88^. Multi-method approaches help mitigate the flaws and biases inherent to any particular method. Piecing together data from different sources improves our understanding of the pandemic^10,16,92,93^. Different methods often reflect different approaches to answering the same question, and thus may produce conflicting estimates. Rather than identifying any single “best” method, multi-method approaches combine diverse sources to produce a collective estimate that is typically more accurate than estimates from individual models. Combining estimates minimizes the pitfalls of relying on any particular individual model, and it can offset statistical bias, potentially canceling out overestimation and underestimation^94–96^. More broadly, multi-method approaches reflect an epistemic commitment to diverse viewpoints^97^. They highlight how the voice of diverse stakeholders may be critical to establishing the ground truth^98^. This is especially relevant in the context of COVID-19 where considerable debate exists about officially reported mortality figures^3,12,19,99–103,119^. Next, we briefly describe various methods used in this study to estimate COVID-19-related excess deaths in Pune. All methods were carried out in accordance with relevant guidelines and regulations. All experimental protocols were approved by Carnegie Mellon University’s Institutional Review Board (Registration No.: IRB00000352).

### Statistical modeling with pre-pandemic all-cause mortality data

To estimate COVID-19-related excess mortality, researchers conventionally use various statistical modeling techniques ranging from simple averaging and linear regression to more sophisticated methods such as Monte-Carlo simulations, Poisson models, and other machine learning models^4,10,11,14,87,104^. Other researchers have estimated excess deaths by extrapolating from more traditional epidemiological measures such as serosurveillance data, infection fatality rate, the overall population’s susceptibility to the virus, the protection offered by vaccination, and the chances of reinfection^10,16,87,106^. Most statistical and epidemiological models computed excess deaths by estimating the number of expected deaths based on pre-pandemic trends^5^. However, different models varied widely in their assumptions and choice of relevant real-world parameters. Some researchers used simple averaging techniques to establish a baseline of expected all-cause mortality^18,87^. Although useful, such simple approaches lack flexibility and robustness because they ignore real-world factors including seasonality, population growth, and contemporary trends of mortality. Epidemiologists addressed these limitations using more sophisticated methods such as widely adopted Poisson and quasi-Poisson models that include parameters such as population growth, seasonality, and recent temporal trends of mortality^4,7,9,20,24,105^. Such models trace their roots to the “classical” Farrington surveillance algorithm that has been extensively used across diverse public health settings over the past three decades^20,106–108^. This approach remains a reference point for many of the improved and extended Poisson-related models that have since been developed^109,110^.

**Figure 2:**
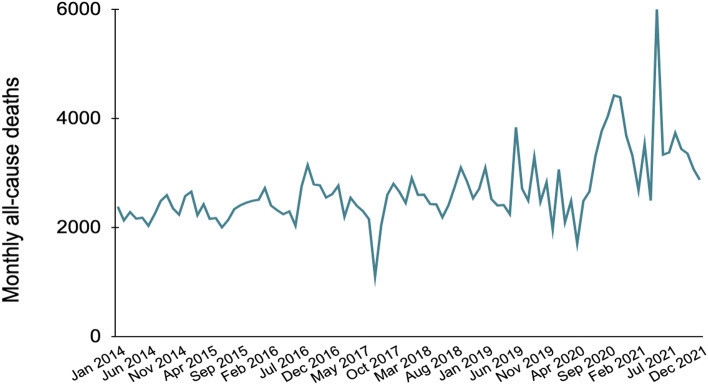
Officially reported monthly all-cause deaths in Pune from January 2014 through December 2021.

Our three statistical models used a dataset about monthly all-cause mortality in Pune from January 2014 through December 2021 (Fig. 2). This dataset was provided to the Jnana Prabodhini Foundation by the Pune Knowledge Cluster, a national-level Science and Innovation Cluster set up by the Office of the Principal Scientific Advisor, Government of India^91^. A formal memorandum of understanding (MoU) of institutional collaboration was signed between the Jnana Prabodhini Foundation and the Pune Knowledge Cluster to ensure responsible data-sharing and upholding privacy standards. The Pune Knowledge Cluster ultimately obtained this dataset from the Pune Municipal Corporation Health Office’s death certificate registration data. Besides estimating excess deaths (Eq. 1), we also computed the undercount factor, the ratio of excess deaths to officially reported COVID-19 death figures (Eq. 2)^17^. ﻿Next, we describe the three statistical models we used in this study.

1 $$excess\; deaths = total \; reported\; deaths - expected\; deaths$$

2 $$undercount\; factor = \frac{excess \;deaths }{{reported \;\text{COVID-19} \;deaths}}$$

### Simple average model

We used a simple nonparametric model^18,87^ to compute COVID-19-related excess deaths (Eq. 3). The expected deaths for each month from March 2020 to December 2021 were calculated as the mean number of total deaths recorded during that month for the previous six years. We also calculated the associated 95% prediction intervals [μ ± *Z*σ] where μ is the mean expected estimate and σ is the standard deviation around the predicted estimate. We set *Z* = 1.96, the 97.5th percentile of a standard normal distribution. Negative values, where observed counts were below the expected thresholds, were set to zero. This method assumes that the number of deaths is effectively constant over time and that the underlying data are independent and identically distributed (i.i.d.). See Supporting Information for further methodological details and an evaluation of model assumptions.

3 $$expected\; deaths = \frac{1}{6}(M_{2014} + M_{2015} + M_{2016} + M_{2017} + M_{2018} + M_{2019} )$$

where *M*_*i*_ is the number of deaths in month *M* of year *i*

### Farrington surveillance algorithm

We implemented the Farrington surveillance algorithm^20^, a quasi-Poisson regression model that accounts for seasonality (Eq. 4), to compute the expected deaths for each month from March 2020 to December 2021. This model was implemented using the *surveillance* package in the R programming language^105,111^. As is standard practice, the lower bound for the margin of error of the Farrington surveillance algorithm was computed using a one-sided 95% prediction interval. The upper bound was computed using average expected deaths. Negative values, where observed counts were below the expected thresholds, were set to zero^9^. This method assumes that the number of deaths is effectively constant over time. See Supporting Information for further methodological details.

4 $$expected\; deaths = e^{{\left( {\alpha + \beta \cdot M} \right)}}$$

where ɑ and β account for a seasonal variation in deaths, and *M* is measured in months.

### Overdispersed Poisson model

We implemented an overdispersed Poisson model that accounts for population growth in addition to seasonal variation in deaths (Eq. 5)^88^ to compute the expected deaths for each month from March 2020 to December 2021. This model was implemented using the *excessmort* package in the R programming language^104^. We obtained estimates about Pune’s monthly population from the World Population Review^89^. We report the associated 95% prediction intervals [μ ± *Z*σ] where μ is the mean expected estimate and σ is the standard deviation around the predicted estimate. We set *Z* = 1.96, the 97.5th percentile of a standard normal distribution. Negative values, where observed counts were below the expected thresholds, were set to zero. See Supporting Information for further methodological details and an evaluation of model assumptions.

5 $$expected \;deaths = P_{M} \cdot e^{{(\alpha_{M} + s_{M} )}}$$

where *P*_*M*_ is the population in month *M*, *ɑ*_*M*_ is a gradual trend accounting for the increasing life expectancy, and *s*_*M*_ is a seasonal trend accounting for a seasonal variation in deaths.

### Analyzing media reports about discrepancies between official mortality data and death compensation claims

Governmental bodies across the world including India’s National Disaster Management Authority have implemented ex gratia monetary compensation policies targeted at households who lost family members to COVID-19^101,112^. Such policies often employ liberal definitions of COVID-19 mortality, thus counting some of the COVID-19 deaths that may have been missed for various reasons^3,4,7,8^, such as deaths that had occurred within a month of suffering from COVID-19 as well as the deaths of patients who did not possess positive RT-PCR (reverse transcription*–*polymerase chain reaction) tests, but nevertheless displayed other indicators of likely COVID-19 infection including positive antibody tests and HRCT (high-resolution computed tomography) chest scans^113^. We analyzed reports from the *Times of India*^113^, one of India’s most-circulated daily newspapers, about the number of COVID-19 death compensation claims filed by households that lost family members to COVID-19. We treated this number as the estimated COVID-19-related excess deaths (Eq. 3). We then computed the undercount factor as the ratio between the number of registered COVID-19 death compensation claims and the number of officially reported COVID-19 deaths (Eq. 4). Unlike statistical modeling, our analysis of death compensation claims only provides a point estimate of excess deaths. However, to heuristically estimate the margin of error associated with our point estimate, we further computed undercount factors for other cities in Maharashtra. Together, these cities constitute a fifth of Maharashtra state’s population and almost half of Maharashtra’s urban population. We calculated the standard error for the undercount factors, thus generating a range of plausible undercount factors for cities in Maharashtra [*se* = σ/√*n* where σ is the standard deviation across these cities and *n* is the number of cities]. This standard error was used to compute a 95% confidence interval for Pune [μ ± Z*se] where μ is the estimated undercount factor for Pune. We set *Z* = 1.96, the 97.5th percentile of a standard normal distribution. The lower and upper bounds of this confidence interval were multiplied by the number of reported COVID-19 deaths to compute plausible lower and upper estimates of excess COVID-19-related deaths in Pune. See Supporting Information for alternative heuristics of computing plausible lower and upper estimates of the undercount factor for Pune.

6 $$excess\; deaths = reported \;\text{COVID-19} \;death \;compensation \;claims$$

7 $$undercount \; factor = \frac{ reported \;\text{COVID-19}\; death \;compensation \;claims}{{reported\; \text{COVID-19} \;deaths}}$$

### Wisdom of crowds public surveying

We conducted an online wisdom of crowds survey in Pune to obtain COVID-19-related excess death estimates. Ethics approval for this survey was obtained from Carnegie Mellon University’s Institutional Review Board (Registration No.: IRB00000352). Only adults participated in the survey and completed a digital consent form before proceeding to the survey questionnaire. Thus, we confirm that informed consent was obtained from all participants. We did not collect identifying or potentially identifying information about survey respondents. We deployed the survey from 8 January 2022 to 8 February 2022. Participants responded to the survey hosted on the SurveyMonkey platform (now Momentive) in either Marathi or English. We employed a sample-of-convenience snowball-sampling method and promoted the survey via social media platforms such as WhatsApp and Facebook. 280 adult residents of Pune participated in a COVID-19-related Knowledge, Attitudes, and Practices (KAP) survey (Table S2)^27^. Survey respondents were asked COVID-19-related questions including: “*As of January 1, 2022, there have been 9,117 COVID-19 deaths in Pune during the pandemic. This data is from official government figures released by Pune Municipal Corporation (PMC). What do you think is the true number of COVID-19 deaths in Pune (as of January 1, 2022)? Please choose a number between 0 and 90,000.*” The average cognitive estimate obtained from public surveying, that is, the collective guess about the “true number of COVID-19 deaths” was considered to be the number of excess COVID-19-related deaths (Eq. 5). We computed the undercount factor as the ratio between the collective cognitive estimate of the speculated true number of COVID-19 deaths and the number of officially reported COVID-19 deaths (Eq. 6). We calculated the standard error [*se* = σ/√n] and used it to compute the 95% confidence interval [μ ± Z*se] where we set *Z* = 1.96, the 97.5th percentile of a standard normal distribution.

8 $$excess \;deaths = collective \;cognitive \;estimate \;of\; COVID - 19\; deaths$$

9 $$undercount \;factor = \frac{ collective\; cognitive \;estimate\; of\; COVID - 19 \;deaths}{{reported \;COVID - 19 \;deaths}}$$

### Aggregate estimate

We combined five COVID-19-related excess deaths and undercount factors obtained from different methods: (a) the simple averaging technique, (b) the Farrington surveillance algorithm, (c) the overdispersed Poisson model, (d) analyzing media-reported death compensation claims, and (e) the wisdom of crowds public surveying. We used a simple bootstrap to generate a plausible range of excess deaths and undercount factors for Pune. We first randomly sampled from the distributions generated by each of the five different methods. For all methods except the wisdom of crowds, we conducted sampling assuming a normal distribution. For the wisdom of crowds, we did not have any such assumption and conducted sampling from the raw survey data. We conducted 10,000 iterations of such random sampling with replacement and used the resulting 10,000 means to compute a 95% confidence interval. See Supporting Information for further methodological details.

## Results

**Figure 3:**
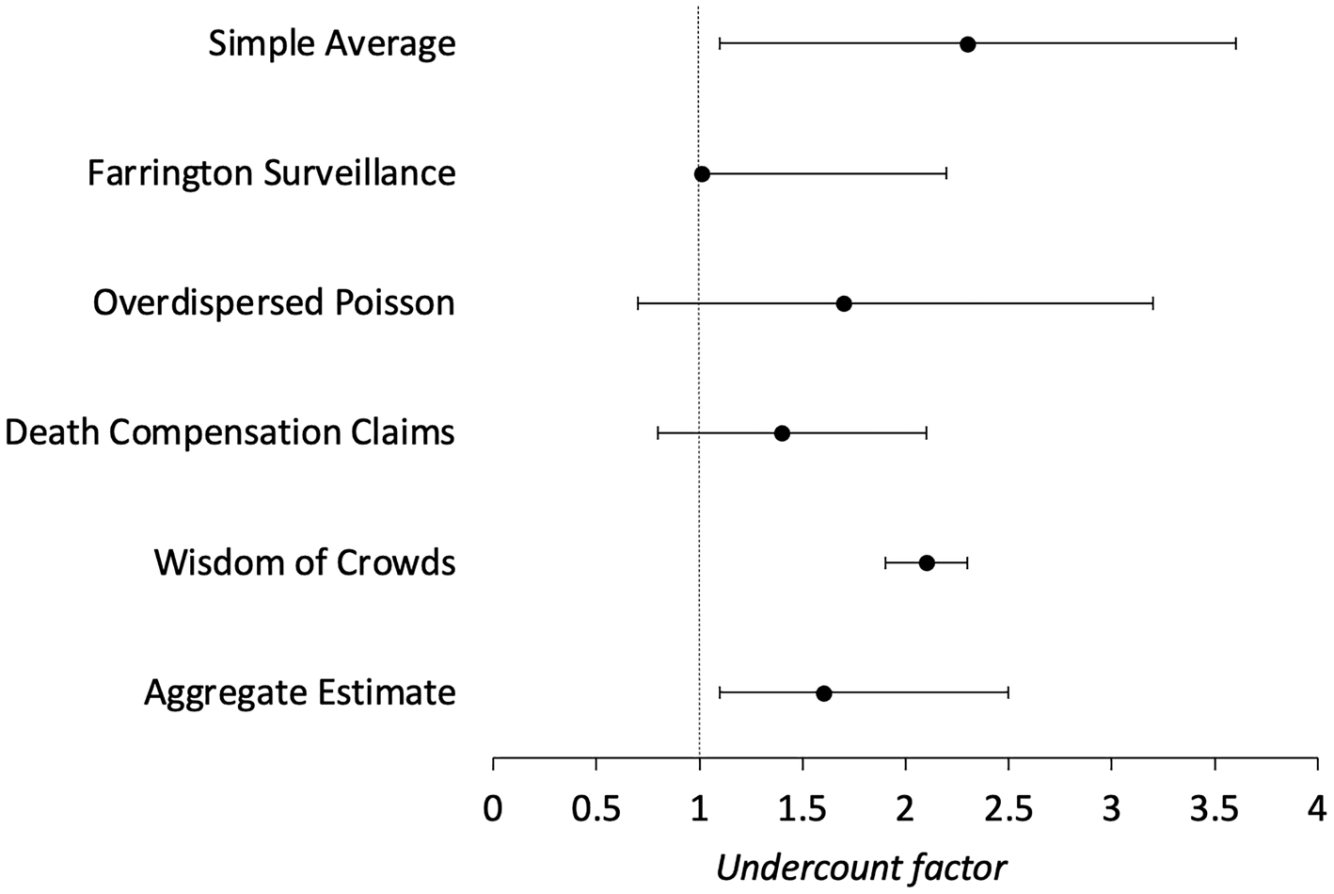
Undercount factor computed from COVID-19-related excess deaths in Pune. The margin of error is the 95% PI for the statistical models: simple average, Farrington surveillance algorithm (one-sided), and overdispersed Poisson model. It is the 95% CI for the analysis of death compensation claims from media reports, the wisdom of crowds public surveying, and the aggregate estimate. An undercount factor of 1 represents an ideal scenario where all estimated excess deaths can be attributed to officially reported COVID-19 mortality.

We used a multi-method approach to compute COVID-19-related excess death estimates in Pune from March 2020 to December 2021 compared to the 74,289 total reported deaths during this time. We also computed the undercount factor in this period, that is, the ratio of estimated excess deaths to the 9,093 officially reported COVID-19 deaths. Table 1 and Fig. 3 present a summary of excess death estimates and undercount factors estimated from all different methods in this study. All estimated expected deaths and excess deaths have been rounded to the nearest 10 to avoid a false sense of precision.

**Table 1:** Estimated expected deaths, excess deaths, and undercount factors during the COVID-19 pandemic in Pune.

Method	Expected deaths	Excess deaths	Undercount factor
Simple average	54,390 (41,230–64,230)	20,490 (10,050–33,050)	2.3 (1.1–3.6)
Farrington surveillance	65,090 (54,390–65,090)	9,200 (9,200–19,900)	1.01 (1.01–2.2)
Overdispersed Poisson	59,110 (45,200–68,300)	15,180 (5,990–29,090)	1.7 (0.7–3.2)
Death compensation claims	*NA*	13,000^a^ (6,910–19,100)	1.4 (0.8–2.1)
Wisdom of crowds	*NA*	18,900 (16,930–20,880)	2.1 (1.9–2.3)
Aggregate estimate	*NA*	14,770 (9,820–22,790)	1.6 (1.1–2.5)

The values in the parentheses are the lower and upper bounds of the margin of error associated with each estimate. The margin of error is the 95% PI for the statistical models: simple average, Farrington surveillance algorithm (one-sided), and overdispersed Poisson model. It is the 95% CI for the analysis of death compensation claims from media reports, the wisdom of crowds public surveying, and the aggregate estimate. ^a^Unlike other cities, the number of death compensation claims filed for Pune based on the media report was approximate^113^.

**Figure 4:**
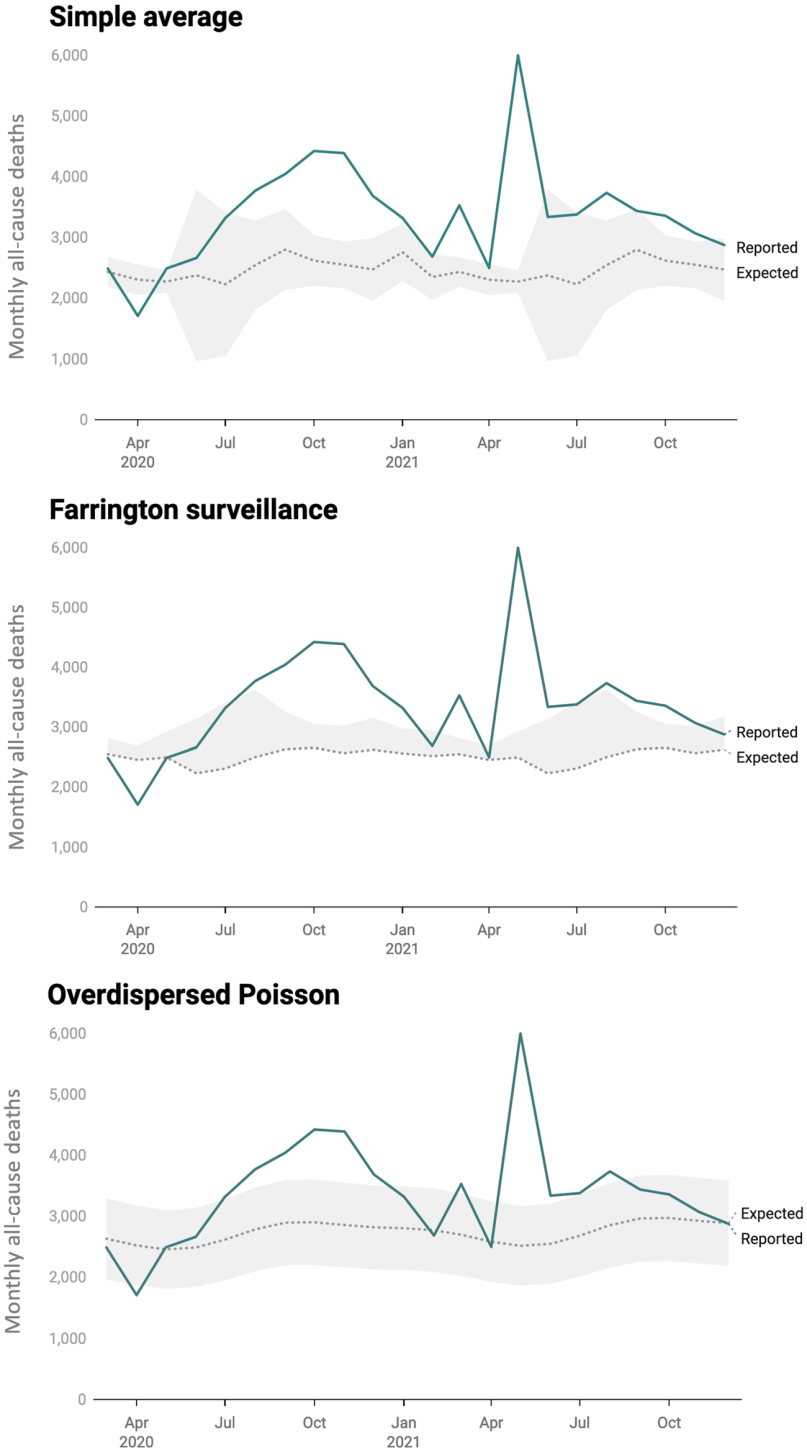
Results from three statistical models: **A**) the simple average model, **B**) the Farrington surveillance algorithm, **C**) and the overdispersed Poisson model. The dotted lines show the expected deaths (estimated from the statistical models) in Pune, the green lines show the officially reported all-cause deaths in Pune, and the gray bands show the 95% PI (one-sided for the Farrington surveillance algorithm).

First, we used three types of statistical models. Based on the pre-pandemic trends, the simple average model estimated 53,790 expected deaths (95% PI: 41,230–64,230). Therefore, the estimated COVID-19-related excess deaths were 20,490 (95% PI: 10,050–33,050) (Fig. 4A). Compared to the estimated excess deaths, the 9,093 officially reported COVID-19 deaths were an undercount of 2.3 (95% PI: 1.1–3.6). However, the simple averaging model did not incorporate seasonal variation in deaths. Accounting for seasonal variation, the Farrington surveillance algorithm estimated 65,090 expected deaths (one-sided 95% PI: 54,390–65,090). Therefore, this method revealed 9,200 estimated excess deaths (one-sided 95% PI: 9,200–19,900) with an undercount factor of 1.01 (one-sided 95% PI: 1.01–2.2) (Fig. 4B). In addition to seasonal variation, the overdispersed Poisson model accounted for population growth and estimated 59,110 expected deaths (one-sided 95% PI: 45,200–68,300), implying 15,180 estimated excess deaths (95% PI: 5,990–29,090) with an undercount factor of 1.7 (95% PI: 0.7–3.2) (Fig. 4C).

Second, we analyzed media reports about discrepancies between official mortality data and the number of COVID-19 death compensation claims filed by the public. As of January 2022, residents of Pune had filed around 13,000 death compensation claims^113^, which served as the estimated COVID-19-related excess deaths in Pune based on media reports. Compared to the officially reported mortality, this figure was an undercount factor of 1.4. Using the same media reports^113^, we additionally computed excess deaths and undercount factors for other major cities in Maharashtra. Table 2 represents a summary of death compensation claims filed at different major cities in Maharashtra and the resultant undercount factors of COVID-19-related excess deaths. Finally, we used the undercount factors from cities in Maharashtra to compute a 95% confidence interval for Pune. Our analysis of media reports about discrepancies between official mortality data and the number of COVID-19 death compensation claims filed by the public point to an estimated 13,000 excess deaths [95% CI: 6,910–19,100] in Pune from March 2020 to January 2022 (Table 1), implying an undercount factor of 1.4 [95% CI: 0.8–2.1].

**Table 2:** Discrepancies between filed death compensation claims and officially reported deaths and estimates of undercount factors during the COVID-19 pandemic in major cities in Maharashtra as of January 2022.

City	Excess COVID-19-related deaths		Undercount factor
	Filed claims	Official deaths	Estimate
Ahmednagar	5,889	1,636	3.6
Aurangabad	5,733	2,329	2.5
Mumbai	17,052	16,581	1.03
Nagpur	12,069	6,055	2.0
Nashik	8,607	4,678	1.8
Pune	13,000^a^	9,093	1.4
Satara	8,194	6,537	1.3
Solapur	5,224	4,152	1.2

^a^Unlike other cities, the number of death compensation claims filed for Pune based on the media report was approximate^114^.

**Figure 5:**
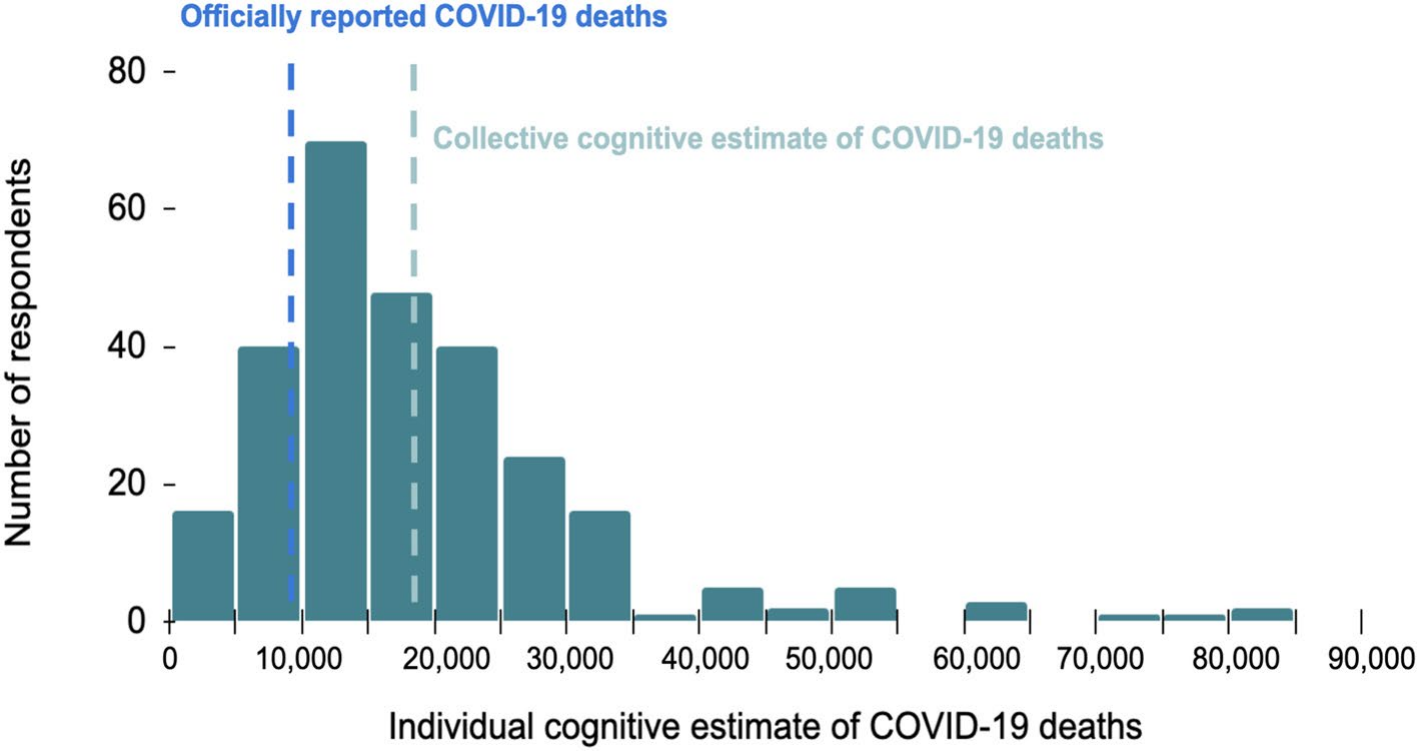
Results from the wisdom of crowds public survey. *N* = 280.

Third, we conducted a wisdom of crowds survey to obtain cognitive estimates about pandemic-associated excess mortality. Cognitive estimates for excess deaths were diverse, with a sixth of survey respondents believing the official COVID-19 numbers were in fact an overestimate (Fig. 5). However, the crowd estimated that the true number of COVID-19 deaths in Pune was 18,900 [95% CI: 16,930–20,880], which served as the estimated COVID-19-related excess deaths. In other words, the crowd estimated an undercount factor of 2.1 [95% CI: 1.9–2.3].

Finally, we used a simple bootstrap to combine estimates from different methods and computed an aggregate estimate of COVID-19-related excess deaths in Pune (Fig. S11). Aggregately, our results estimate 14,770 excess deaths [95% CI: 9,820–22,790] in Pune from March 2020 to December 2021, translating to an undercount factor of 1.6 [95% CI: 1.1–2.5].

## Discussion

In our case study, we computed COVID-19-related excess death estimates for Pune. To our knowledge, this is the first such effort; therefore, our results provide new information that can inform the public health policy of Pune. Using multiple methods, we estimated 14,770 excess deaths [95% CI: 9,820–22,790] in Pune from March 2020 to December 2021, of which 9,093 were officially counted as COVID-19 deaths. We further calculated the undercount factor, a metric that allowed for easy comparison of the differential impact of the pandemic across diverse geographical regions and socioeconomic groups^2,13,21,113^. We estimated an undercount factor of 1.6 [95% CI: 1.1–2.5] for Pune from March 2020 to December 2021. Thus, we estimated excess COVID-19-related deaths were about 60% more than officially recorded. An undercount factor of 1 implies that all the estimated excess deaths can be attributed to officially reported COVID-19 mortality. This represents an ideal scenario where public health infrastructures are robust and resilient enough to maintain complete and high-quality data, even during acute crisis events such as pandemics. However, this ideal scenario was rarely achieved globally and across major Indian cities, where the estimated undercount factors were around three (Table S1)^10,14,15,22,156^. Even some of the world’s best healthcare systems saw undercount factors around 1.5 (Fig. S2 in Supporting Information)^8,113^. Based on our results, Pune’s performance in this regard seems comparable to some of the leading healthcare systems across the world, with its public health data recording infrastructure proving to be fairly robust and resilient during the COVID-19 pandemic^115,116^.

In addition to providing novel public health information about Pune, our main goal was to investigate whether diverse methods of estimating pandemic-related excess deaths provided us with accurate and overlapping statistical estimates. We computed COVID-19-related excess deaths and undercount factors from five different methods: (a) the simple averaging technique, (b) the Farrington surveillance algorithm, (c) the overdispersed Poisson model, (d) analyzing media-reported discrepancies between official mortality data and death compensation claims, and (e) the wisdom of crowds public surveying. Despite their limitations, diverse methods—both conventional and frugal—produced excess deaths estimates and undercount factors that were within the margins of error of each other. Results from all models except from the Farrington surveillance algorithm point towards a similar conclusion about the COVID-19-related undercount factor for Pune. These findings can inform Pune's public health policy—for future pandemics or health crises, decision-makers could assume a worst-case scenario and prepare for up to 2.5 times (upper limit of the 95% confidence interval associated with our aggregate estimate) the reported number of pandemic-caused deaths. Our results reinforce the strength of using multi-method approaches to triangulate the true extent of the impact of the COVID-19 pandemic. By combining conventional and novel frugal methods of estimating pandemic-associated excess mortality in a multi-method approach, we minimized the pitfalls of relying on any particular individual method^86,95–98,117,118^. Our findings can have important implications, especially in resource-constrained settings, where robust and resilient data infrastructures tend to be lacking or limited, and in contexts where considerable debate exists about the underlying ground truth^1–3,12,19,26–28,99–103,119^. Particularly with the COVID-19 pandemic, there are widespread concerns about the accuracy of officially reported COVID-19-related deaths^3–8^. Our study adds to a growing body of COVID-19-related excess mortality literature that emerged in response to such skepticism about the accuracy of officially reported pandemic casualties^3–5,7,9–15,22^. Future research efforts could focus on other untapped frugal alternatives such as analyzing discrepancies between COVID-19 cremation counts and officially reported COVID-19 mortality data^157,158^.  Our preliminary results from this method for Pune suggest consilience with the other methods we employed in our study (Table S3). However, these preliminary results are based on a temporally restricted dataset about COVID-19 cremation counts, and a more complete dataset is needed to ascertain the robustness of this method.

Within our multi-method approach, we employed three conventional statistical and epidemiological models that have been previously widely used to compute COVID-19-related excess mortality. These methods are often considered the gold standard of excess mortality estimation because of their interpretability and inclusion of multiple epidemiologically relevant real-world factors including seasonality, population growth, and contemporary trends of mortality^4–25^. Therefore, our results from these methods represent important benchmarks to examine the effectiveness of the novel frugal methods we used. However, these conventional statistical and epidemiological models rely on high-quality all-cause pre-pandemic data that is only accessible in robust and transparent public health data recording systems. The performance of these models suffers in the absence of such data. One limitation of our study was the low granularity of our dataset; it included only monthly—not weekly or daily—data. Future research efforts can address this limitation by using high-granularity datasets. Additionally, although Pune is estimated to have high pre-pandemic death registration coverage^18,120^, our study did not account for fluctuations in death registration coverage during the COVID-19 pandemic. Future work should use indirect proxy estimates of fluctuations in death registration coverage that can be computed from relevant public health and demographic data such as birth registration coverage, the incidence of traffic accidents, and surveillance of other infectious diseases such as AIDS and tuberculosis (Table S4)^18,121–124^.

Two of the statistical models we used: a) the simple averaging technique and b) the Farrington surveillance algorithm did not incorporate underlying population data, and therefore can be readily deployed when these data are non-existent or difficult to obtain due to monetary, bureaucratic, and time constraints. An additional strength of the simple averaging technique is its ease of implementation. This method does not require computer programming knowledge, thus increasing its potential for widespread applicability in low-resource and data-scarce settings. Both the simple averaging technique and the Farrington surveillance algorithm assumed that the pre-pandemic number of deaths was effectively constant over time. We assessed this assumption for both models (see Supporting Information). Even though there was a slight yet significant increase in mortality over time (Fig. S4), both models showed relatively robust performance despite this violated assumption (Fig. S5, Fig. S6, Fig. S7, and Fig. S8). Robust model performance depended upon the amount of underlying data used—both models required monthly data across at least four years. The overdispersed Poisson model incorporated underlying population data to account for fluctuations in mortality rates over time and thus did not assume that the pre-pandemic number of deaths was effectively constant over time. It also accounted for sustained indirect effects that both the simple averaging technique and the Farrington surveillance algorithm lacked the power to detect^88^, thereby offering more flexibility and robustness compared to these two models. Finally, the overdispersed Poisson nature of this model allowed it to capture more variance than predicted by a Poisson model. This makes it well-suited to our dataset of monthly reported all-cause mortality (mean = 2,687; variance = 418,337).

In addition to using statistical and epidemiological models, we also analyzed media reports about discrepancies between official mortality data and death compensation claims. To our knowledge, our study is the first effort to use this frugal method to estimate pandemic-associated excess deaths. The analyses in this method were possible only because of the availability of data about death compensation claims filed by the public under India’s ex gratia monetary compensation policy that employed a liberal interpretation of pandemic-associated mortality^101,113,159,160^. However, this policy may have led to somewhat inaccurate estimates of excess mortality due to the submission of fraudulent documents or the double counting of deaths in neighboring jurisdictions^ 125,159,160^. Nonetheless, this frugal method remains an important component of multi-method approaches to estimating excess COVID-19-related deaths, given the checks and balances implemented by the government to ensure accurate relief disbursement^113^. Future research should use disaggregated and officially verified ex gratia death compensation data to compute more precise estimates of pandemic-associated excess mortality.

Finally, we examined the effectiveness of another frugal method—the wisdom of crowds approach—to estimate COVID-19-related excess mortality. Although this approach has been widely used across multiple real-world domains before, including during the COVID-19 pandemic^37–86,126,127^, to our knowledge, this frugal method has not yet been used to estimate COVID-19-related excess mortality. Therefore, our study provides a novel confirmation of the potential of the wisdom of crowds approach as a complementary tool of frugal fact-finding. However, the results from our wisdom of crowds public survey should be interpreted with caution, because collective cognitive estimates may be biased, sometimes resulting in herding, mob mentality, informational echo chambers, and widespread proliferation of unscientific opinions^128–137^. Nonetheless, these limitations can be overcome by integrating findings from judgment, decision-making, behavioral economics, and cognitive science that highlight how domain-general psychophysical representations and Bayesian mechanisms may account for many of the systematic mistakes observed in cognitive estimation across many real-world contexts^137–149,151^. These findings suggest that domain-general processes account for many of the quirks of human estimation, judgment, and decision-making. Accounting for such general psychophysical factors and other cognitive biases can greatly improve the accuracy, robustness, and effectiveness of the wisdom of crowds approach^150^. For example, in our study, we were able to partially mitigate the biases introduced due to social and peer influence^127,128,130,151^ by conducting an online, anonymous public survey. In addition to being a non-WEIRD (*Western, Educated, Industrialized, Rich, and Democratic*) population^152^, our survey sample of adult residents from Pune was diverse in terms of gender, age, native language, occupation, socioeconomic status, and COVID-19 infection history (Table S2). These study participants also displayed heterogeneous COVID-19-related beliefs and behaviors. Thus, the diversity, decentralization, and independence of opinions^126^ in our sample may have mitigated some of the inaccuracies stemming from demand characteristics and response biases. In our future work, we plan to explore how diverse COVID-19-related psychological perceptions influence cognitive estimates about COVID-19-related deaths, thus adding to a rapidly growing literature about cognitive estimation and the wisdom of crowds.

Our findings confirm that, like most other places, officially reported COVID-19 mortality in Pune was an underestimate. These findings highlight the limitations of public health infrastructures in capturing plentiful, high-quality, and timely data during unpredictable black swan events such as the COVID-19 pandemic^153^. To address these limitations, strong health data systems are needed to inform healthcare utilization planning, resource allocation, and policymaking to ensure healthy living and promote well-being for all (UN Sustainable Development Goal 3)^154^. Robust data systems also permit post-mortem evaluations of pandemic mitigation measures including vaccinations and public lockdowns^156^. To prepare for future pandemics, resilient public health systems require sustained material investments in vital infrastructure and medical equipment, as well as the availability of credible, open-source, and high-quality data (UN Sustainable Development Goal 17.19)^154^. The success of these initiatives will depend on both long-term material investments in vital infrastructure and medical equipment, as well as the availability and abundance of credible, open-source, high-quality data. Therefore, governments, think tanks, research universities, non-profits, industry actors, the media, and other relevant stakeholders have an onus to build and maintain robust data collection and storage infrastructures. This will support wider aims of sensitive societal governance, public accountability, and memorialization of one of the largest public health crises the world has collectively faced in over a century^1,2^.

### Supplementary Information

Supplementary Information.

## Data Availability

All data generated or analyzed during this study are included in this published article and its supplementary information files.

## References

[1] Jnana Prabodhini Foundation. Pandemic, Punekars, and Policy. Accessed from: https://www.youtube.com/watch?v=UTwAH8wHG3Y. Accessed 15 October 2022.

[2] Whittaker C, Walker PG, Alhaffar M, Hamlet A, Djaafara BA, Ghani A, Ferguson N, Dahab M, Checchi F, Watson OJ (2021). Under-reporting of deaths limits our understanding of true burden of covid-19. BMJ.

[3] Adam D (2022). COVID’s true death toll: much higher than official records. Nature..

[4] Knutson V, Aleshin-Guendel S, Karlinsky A, Msemburi W, Wakefield J (2023). Estimating global and country-specific excess mortality during the COVID-19 pandemic. Ann. Appl. Stat..

[5] Ritchie, H., Mathieu, E., Rodés-Guirao, L., Appel, C., Giattino, C., Ortiz-Ospina, E., Hasell, J., Macdonald, B., Beltekian, D. & Roser, M. Coronavirus pandemic (COVID-19). Our world in data (2020).

[6] Zimmermann LV, Salvatore M, Babu GR, Mukherjee B (2021). Estimating COVID-19-related mortality in India: An epidemiological challenge with insufficient data. Am. J. Public Health.

[7] Islam N, Shkolnikov VM, Acosta RJ, Klimkin I, Kawachi I, Irizarry RA, Alicandro G, Khunti K, Yates T, Jdanov DA, White M (2021). Excess deaths associated with covid-19 pandemic in 2020: age and sex disaggregated time series analysis in 29 high income countries. BMJ.

[8] Wang H, Paulson KR, Pease SA, Watson S, Comfort H, Zheng P, Aravkin AY, Bisignano C, Barber RM, Alam T, Fuller JE (2022). Estimating excess mortality due to the COVID-19 pandemic: A systematic analysis of COVID-19-related mortality, 2020–21. The Lancet..

[9] Centers for Disease Control and Prevention (CDC). Excess Deaths Associated with COVID-19. 2021 Nov. Accessed from: https://www.cdc.gov/nchs/nvss/vsrr/covid19/excess_deaths.htm

[10] Jha P, Deshmukh Y, Tumbe C, Suraweera W, Bhowmick A, Sharma S, Novosad P, Fu SH, Newcombe L, Gelband H, Brown P (2022). COVID mortality in India: National survey data and health facility deaths. Science..

[11] Leffler CT, Das S, Yang E, Konda S (2022). Preliminary analysis of excess mortality in India during the COVID-19 pandemic. Am. J. Trop. Med. Hygiene..

[12] Gamio, S. & Glanz, J. Just how big could India’s true covid toll be? The New York Times (2021).

[13] Rossen LM, Branum AM, Ahmad FB, Sutton PD, Anderson RN (2021). Notes from the field: Update on excess deaths associated with the COVID-19 pandemic—United States, January 26, 2020–February 27, 2021. Morbid. Mortal. Wkly. Rep..

[14] The Economist, Solstad S. The pandemic’s true death toll. The Economist. 2021 May.

[15] Acosta E (2022). Global estimates of excess deaths from COVID-19. Nature..

[16] Anand, A., Sandefur, J. & Subramanian, A. Three new estimates of India’s all-cause excess mortality during the COVID-19 pandemic. Center for Global Development. (2021).

[17] Banaji, M. Covid-19: What data about excess deaths reveals about Mumbai’s class divide. Scroll. (2021).

[18] Banaji, M. Estimating COVID-19 infection fatality rate in Mumbai during 2020. medRxiv. 2021 Apr 10:2021–04.

[19] Biswas, S. Why India’s real COVID toll may never be known. *BBC News*. (2022).

[20] Farrington CP, Andrews NJ, Beale AD, Catchpole MA (1996). A statistical algorithm for the early detection of outbreaks of infectious disease. J. R. Stat. Soc. Ser. A (Stat. Soc.)..

[21] Karlinsky, A. & Kobak, D. Tracking excess mortality across countries during the COVID-19 pandemic with the World Mortality Dataset. *Elife*. (2021). 10.7554/eLife.69336 PMC8331176 34190045

[22] Msemburi W, Karlinsky A, Knutson V, Aleshin-Guendel S, Chatterji S, Wakefield J (2022). The WHO estimates of excess mortality associated with the COVID-19 pandemic. Nature..

[23] Parkin, B., Singh, J., Findlay, S. & Burn-Murdoch, J. India’s devastating second wave:‘It is much worse this time.’. *Financial Times* (2021).

[24] Santos-Burgoa C, Sandberg J, Suárez E, Goldman-Hawes A, Zeger S, Garcia-Meza A, Pérez CM, Estrada-Merly N, Colón-Ramos U, Nazario CM, Andrade E (2018). Differential and persistent risk of excess mortality from Hurricane Maria in Puerto Rico: a time-series analysis. The Lancet Planetary Health..

[25] Vandoros S (2020). Excess mortality during the Covid-19 pandemic: Early evidence from England and Wales. Soc. Sci. Med..

[26] Akhlaq A, McKinstry B, Muhammad KB, Sheikh A (2016). Barriers and facilitators to health information exchange in low-and middle-income country settings: a systematic review. Health Policy Plann..

[27] Jnana Prabodhini Foundation. Pandemic, Punekars, and Perceptions: Preliminary findings of a COVID-19-related Knowledge, Attitudes, Practices, and Wisdom survey. 2021 Jul. Accessed from: jnanaprabodhinifoundation.org/analytics.

[28] von Clausewitz, C. On the Nature of War. In On War. 2008 Sep 2 (pp. 73–124). Princeton University Press.

[29] Mwananyanda L, Gill CJ, MacLeod W, Kwenda G, Pieciak R, Mupila Z, Lapidot R, Mupeta F, Forman L, Ziko L, Etter L (2021). Covid-19 deaths in Africa: prospective systematic postmortem surveillance study. BMJ.

[30] Djaafara BA, Whittaker C, Watson OJ, Verity R, Brazeau NF, Widyastuti W, Oktavia D, Adrian V, Salama N, Bhatia S, Nouvellet P (2020). Quantifying the dynamics of COVID-19 burden and impact of interventions in Java, Indonesia. MedRxiv..

[31] Endris, B. S., Saje, S. M., Metaferia, Z. T., Sisay, B. G., Afework, T., Mengistu, Y. G., Fenta, E. H., Gebreyesus, S. H., Petros, A., Worku, A. & Seman, Y. Excess mortality in the face of COVID-19: evidence from Addis Ababa mortality surveillance program. [Preprint.]. 10.2139/ssrn.3787447.

[32] Koum Besson ES, Norris A, Bin Ghouth AS, Freemantle T, Alhaffar M, Vazquez Y, Reeve C, Curran PJ, Checchi F (2021). Excess mortality during the COVID-19 pandemic: A geospatial and statistical analysis in Aden governorate, Yemen. BMJ Glob. Health..

[33] Morris, J. What does USA Group Term Life Insurance Report say about Young Adult Excess Deaths in Fall 2021?. COVID-19 Data Science. 2022 Aug 25. https://www.covid-datascience.com/post/what-does-usa-group-term-life-insurance-report-say-about-young-adult-excess-deaths-in-fall-2021

[34] The Reporters Collective. Available from: https://www.reporters-collective.in. 2021.

[35] Watson OJ, Alhaffar M, Mehchy Z, Whittaker C, Akil Z, Brazeau NF, Cuomo-Dannenburg G, Hamlet A, Thompson HA, Baguelin M, FitzJohn RG (2021). Leveraging community mortality indicators to infer COVID-19 mortality and transmission dynamics in Damascus, Syria. Nat. Commun..

[36] Onuah F (2020). At least half of mystery deaths in Nigeria’s Kano due to COVID-19. Reuters.

[37] Bullard SE, Fein D, Gleeson MK, Tischer N, Mapou RL, Kaplan E (2004). The Biber cognitive estimation test. Arch. Clin. Neuropsychol..

[38] Galton F (1907). Vox populi (the wisdom of crowds). Nature..

[39] Shallice T, Evans ME (1978). The involvement of the frontal lobes in cognitive estimation. Cortex..

[40] Delaloye C, Moy G, Baudois S, De Bilbao F, Remund CD, Hofer F, Paquier CR, Weber K, Urben S, Giannakopoulos P (2009). The contribution of aging to the understanding of the dimensionality of executive functions. Arch. Gerontol. Geriatr..

[41] Fisk JE, Sharp CA (2004). Age-related impairment in executive functioning: Updating, inhibition, shifting, and access. J. Clin. Exp. Neuropsychol..

[42] Jurado MB, Rosselli M (2007). The elusive nature of executive functions: A review of our current understanding. Neuropsychol. Rev..

[43] Spreen, O. General intellectual ability and assessment of premorbid intelligence. A compendium of neuropsychological tests. 43–135 (1998).

[44] Stuss DT, Alexander MP (2000). Executive functions and the frontal lobes: a conceptual view. Psychol. Res..

[45] Stuss DT, Levine B (2002). Adult clinical neuropsychology: lessons from studies of the frontal lobes. Annu. Rev. Psychol..

[46] Appollonio IM, Russo A, Isella V, Forapani E, Villa ML, Piolti R, Frattola L (2003). Cognitve estimation: comparison of two tests in nondemented parkinsonian patients. Neurol. Sci..

[47] Axelrod BN, Millis SR (1994). Preliminary standardization of the cognitive estimation test. Assessment..

[48] Bisbing TA, Olm CA, McMillan CT, Rascovsky K, Baehr L, Ternes K, Irwin DJ, Clark R, Grossman M (2015). Estimating frontal and parietal involvement in cognitive estimation: a study of focal neurodegenerative diseases. Front. Hum. Neurosci..

[49] Della Sala S, MacPherson SE, Phillips LH, Sacco L, Spinnler H (2004). The role of semantic knowledge on the cognitive estimation task: Evidence from Alzheimer’s disease and healthy adult aging. J. Neurol..

[50] Goldstein FC, Green J, Presley RM, O'Jile J (1996). Cognitive estimation in patients with Alzheimer's disease. Neuropsychiatry Neuropsychol. Behav. Neurol..

[51] Leng NR, Parkin AJ (1988). Double dissociation of frontal dysfunction in organic amnesia. Br. J. Clin. Psychol..

[52] Levinoff EJ, Phillips NA, Verret L, Babins L, Kelner N, Akerib V, Chertkow H (2006). Cognitive estimation impairment in Alzheimer disease and mild cognitive impairment. Neuropsychology..

[53] Shoqeirat MA, Mayes A, MacDonald C, Meudell P, Pickering A (1990). Performance on tests sensitive to frontal lobe lesions by patients with organic amnesia: Leng & Parkin revisited. Br. J. Clin. Psychol..

[54] Spencer RJ, Johnson-Greene D (2009). The Cognitive Estimation Test (CET): Psychometric limitations in neurorehabilitation populations. J. Clin. Exp. Neuropsychol..

[55] Taylor R, O'Carroll R (1995). Cognitive estimation in neurological disorders. Br. J. Clin. Psychol..

[56] Wagner GP, MacPherson SE, Parente MA, Trentini CM (2011). Cognitive estimation abilities in healthy and clinical populations: the use of the Cognitive Estimation Test. Neurol. Sci..

[57] Ashkenazi S, Tsyganov Y (2017). The Cognitive Estimation Task is nonunitary: Evidence for multiple magnitude representation mechanisms among normative and ADHD college students. J. Numer. Cogn..

[58] Cokely ET, Galesic M, Schulz E, Ghazal S, Garcia-Retamero R (2012). Measuring risk literacy: The Berlin numeracy test. Judgm. Decis. Mak..

[59] Harel BT, Cillessen AH, Fein DA, Bullard SE, Aviv A (2007). It takes nine days to iron a shirt: The development of cognitive estimation skills in school age children. Child Neuropsychol..

[60] Huizinga MM, Carlisle AJ, Cavanaugh KL, Davis DL, Gregory RP, Schlundt DG, Rothman RL (2009). Literacy, numeracy, and portion-size estimation skills. Am. J. Prev. Med..

[61] Liss M, Fein D, Bullard S, Robins D (2000). Brief report: Cognitive estimation in individuals with pervasive developmental disorders. J. Autism Dev. Disord..

[62] Siegler RS, Booth JL (2004). Development of numerical estimation in young children. Child Dev..

[63] Asch SE (1956). Studies of independence and conformity: I. A minority of one against a unanimous majority. Psychol. Monogr. Gen. Appl..

[64] Hause, P. The limitations of KAP surveys. Social research in developing countries. 65–69 (1993).

[65] Kahneman, D., Slovic, S. P., Slovic, P. & Tversky, A. editors. Judgment under uncertainty: Heuristics and biases. Cambridge University Press (1982).

[66] Manski CF (2004). Measuring expectations. Econometrica..

[67] Atanasov P, Rescober P, Stone E, Swift SA, Servan-Schreiber E, Tetlock P, Ungar L, Mellers B (2017). Distilling the wisdom of crowds: Prediction markets vs prediction polls. Manag. Sci..

[68] Berg JE, Nelson FD, Rietz TA (2008). Prediction market accuracy in the long run. Int. J. Forecast..

[69] Bruine de Bruin W, Galesic M, Bååth R, de Bresser J, Hall L, Johansson P, Strandberg T, van Soest A (2022). Asking about social circles improves election predictions even with many political parties. Int. J. Public Opin. Res..

[70] Chalmers J, Kaul A, Phillips B (2013). The wisdom of crowds: Mutual fund investors’ aggregate asset allocation decisions. J. Bank. Finance.

[71] Epp DA (2017). Public policy and the wisdom of crowds. Cogn. Syst. Res..

[72] Galesic M, Bruine de Bruin W, Dalege J, Feld SL, Kreuter F, Olsson H, Prelec D, Stein DL, van Der. (2021). Human social sensing is an untapped resource for computational social science. Nature..

[73] Galesic M, Bruine de Bruin W, Dumas M, Kapteyn A, Darling JE, Meijer E (2018). Asking about social circles improves election predictions. Nat. Hum. Behav..

[74] Graefe A (2014). Accuracy of vote expectation surveys in forecasting elections. Public Opin. Q..

[75] Olsson, H., Bruine de Bruin, W., Galesic, M. & Prelec, D. Election polling is not dead: A Bayesian bootstrap method yields accurate forecasts (2021). 10.31219/osf.io/nqcgs.

[76] Spann M, Skiera B (2003). Internet-based virtual stock markets for business forecasting. Manag. Sci..

[77] Tetlock, P. E. & Gardner, D. Superforecasting: The art and science of prediction. Random House (2016).

[78] Christakis NA, Fowler JH (2010). Social network sensors for early detection of contagious outbreaks. PLoS ONE..

[79] Polgreen PM, Nelson FD, Neumann GR, Weinstein RA (2007). Use of prediction markets to forecast infectious disease activity. Clin. Infect. Dis..

[80] Rodríguez, A., Kamarthi, H., Agarwal, P., Ho, J., Patel, M., Sapre, S. & Prakash, B. A. Data-centric epidemic forecasting: A survey. arXiv preprint arXiv:2207.09370. (2022).

[81] Bruine de Bruin W, Parker AM, Galesic M, Vardavas R (2019). Reports of social circles’ and own vaccination behavior: A national longitudinal survey. Health Psychol..

[82] Bracher J, Wolffram D, Deuschel J, Görgen K, Ketterer JL, Ullrich A, Abbott S, Barbarossa MV, Bertsimas D, Bhatia S, Bodych M (2021). A pre-registered short-term forecasting study of COVID-19 in Germany and Poland during the second wave. Nat. Commun..

[83] McDonald DJ, Bien J, Green A, Hu AJ, DeFries N, Hyun S, Oliveira NL, Sharpnack J, Tang J, Tibshirani R, Ventura V (2021). Can auxiliary indicators improve COVID-19 forecasting and hotspot prediction?. Proc. Natl. Acad. Sci..

[84] Recchia G, Freeman AL, Spiegelhalter D (2021). How well did experts and laypeople forecast the size of the COVID-19 pandemic?. PLoS ONE.

[85] Sell TK, Warmbrod KL, Watson C, Trotochaud M, Martin E, Ravi SJ, Balick M, Servan-Schreiber E (2021). Using prediction polling to harness collective intelligence for disease forecasting. BMC Public Health.

[86] Taylor KS, Taylor JW (2022). Interval forecasts of weekly incident and cumulative COVID-19 mortality in the United States: A comparison of combining methods. PLoS ONE..

[87] Banaji M, Gupta A (2022). Estimates of pandemic excess mortality in India based on civil registration data. PLOS Glob. Public Health..

[88] Acosta RJ, Irizarry RA (2022). A flexible statistical framework for estimating excess mortality. Epidemiology..

[89] World Population Review. Pune Population 2022. Dec 2022. Accessed from: https://worldpopulationreview.com/world-cities/pune-population

[90] Mundhe N (2019). Identifying and mapping of slums in Pune city using geospatial techniques. Int. Arch. Photogram. Remote Sens. Spat. Inf. Sci..

[91] Pune Knowledge Cluster. All-cause mortality in the Pune Municipal Corporation from 2014 to 2021. Mar 2022. Note—this dataset was generously provided to the Jnana Prabodhini Foundation by the Pune Knowledge Cluster. Accessed from: https://www.pkc.org.in/

[92] Malani, A. & Ramachandran, S. Using household rosters from survey data to estimate all-cause mortality during Covid in India. National Bureau of Economic Research (2021).

[93] McCabe, R., Whittaker, C., Sheppard, R. J., Abdelmagid, N., Ahmed, A., Alabdeen, I. Z., Brazeau, N. F., Abd Elhameed, A. E., Bin-Ghouth, A. S., Hamlet, A. & AbuKoura, R. Alternative epidemic indicators for COVID-19: a model-based assessment of COVID-19 mortality ascertainment in three settings with incomplete death registration systems. medRxiv. 2023:2023–01. 10.1126/sciadv.adg7676 PMC10256151 37294754

[94] Panovska-Griffiths J (2020). Coronavirus: we’ve had Imperial. Oxford and many more models–554 but none can have all the answers. The Conversation.

[95] Roberts, S. All together now: The most trustworthy covid-19 model is an ensemble. MIT Technology Review (2021).

[96] Claeskens G, Magnus JR, Vasnev AL, Wang W (2016). The forecast combination puzzle: A simple theoretical explanation. Int. J. Forecast..

[97] Bohman J (2006). Deliberative democracy and the epistemic benefits of diversity. Episteme..

[98] Cort JE (2000). " Intellectual Ahiṃsā" revisited: Jain tolerance and intolerance of others. Philos. East West..

[99] Choudhary, A. A. Supreme Court: Official Covid toll stats ‘not true’, so don’t deny ex-gratia. The Times of India. (2022).

[100] Ministry of Health and Family Welfare. Excess Mortality Estimates by WHO. 2022 May. Accessed from: https://pib.gov.in/PressReleasePage.aspx?PRID=1823012

[101] Ministry of Health and Family Welfare. Hon’ble Supreme Court fixes timelines for filing of claims for payment of ex-gratia assistance to families of COVID-19 deceased. 2022 Apr. Accessed from: https://pib.gov.in/PressReleasePage.aspx?PRID=1815545

[102] Ministry of Health and Family Welfare. In response to New York Times article titled “India Is Stalling the WHO’s Efforts to Make Global Covid Death Toll Public” dated 16th April, 2022. 2022 Apr. Accessed from: https://pib.gov.in/PressReleasePage.aspx?PRID=1817436

[103] Press Trust of India. India's top health experts question WHO report on excess Covid deaths, term it untenable. The Times of India. 2022 May 6.

[104] Acosta, R. J. & Irizarry, R. A. excessmort: Excess Mortality. 2021. Available from: https://CRAN.R-project.org/package=excessmort

[105] Höhle, M., Meyer, S., Paul, M., Held, L., Burkom, H., Correa, T., Hofmann, M., Lang, C., Manitz, J., Riebler, A., Bove, D., Salmon, M., Schumacher, D., Steiner, S., Virtanen, M., Wei, W., Wimmer, V., R Core Team. Surveillance: Temporal and Spatio-Temporal Modeling and Monitoring of Epidemic Phenomena. 2022. Available from: https://cran.r-project.org/web/packages/surveillance

[106] Wu, J., Mafham, M., Mamas, M. A., Rashid, M., Kontopantelis, E., Deanfield, J. E., de Belder, M. A. & Gale, C. P. Place and underlying cause of death during the COVID-19 pandemic: retrospective cohort study of 3.5 million deaths in England and Wales, 2014 to 2020. InMayo Clinic Proceedings 2021 Apr 1 (Vol. 96, No. 4, pp. 952–963). Elsevier. 10.1016/j.mayocp.2021.02.007 PMC7885692 33714592

[107] Al Wahaibi A, Al-Maani A, Alyaquobi F, Al Harthy K, Al-Jardani A, Al Rawahi B, Al-Abri S (2021). Effects of COVID-19 on mortality: A 5-year population-based study in Oman. Int. J. Infect. Dis..

[108] Kawashima T, Nomura S, Tanoue Y, Yoneoka D, Eguchi A, Ng CF, Matsuura K, Shi S, Makiyama K, Uryu S, Kawamura Y (2021). Excess all-cause deaths during coronavirus disease pandemic, Japan, January–May 2020. Emerg. Infect. Dis..

[109] Noufaily A, Enki DG, Farrington P, Garthwaite P, Andrews N, Charlett A (2013). An improved algorithm for outbreak detection in multiple surveillance systems. Stat. Med..

[110] Höhle M, Paul M (2008). Count data regression charts for the monitoring of surveillance time series. Comput. Stat. Data Anal..

[111] Meyer, S., Held, L. & Höhle, M. Spatio-temporal analysis of epidemic phenomena using the R package surveillance. arXiv preprint arXiv:1411.0416. (2014).

[112] National Disaster Management Authority of India. Public Notice about COVID-19. 2022. Accessed from: https://ndma.gov.in/sites/default/files/PDF/Public_notice_COVID19_eng.pdf

[113] Debroy, S., Jain, B. & Nambiar, N. Pune clears Covid aid for 16,500 victims, 4000 more to get relief. The Times of India. (2022).

[114] International Institute for Population Sciences (IIPS). 2021. National Family Health Survey (NFHS-5), India, 2019–21: Maharashtra. Mumbai: IIPS.

[115] Bogam P, Joshi A, Nagarkar S, Jain D, Gupte N, Shashidhara LS, Monteiro JM, Mave V (2022). Burden of COVID-19 and case fatality rate in Pune, India: An analysis of the first and second wave of the pandemic. IJID Reg..

[116] Mave V, Shaikh A, Monteiro JM, Bogam P, Pujari BS, Gupte N (2022). Association of national and regional lockdowns with COVID-19 infection rates in Pune, India. Sci. Rep..

[117] Bekhet AK, Zauszniewski JA (2012). Methodological triangulation: An approach to understanding data. Nurse Res..

[118] Wilson, E. O. Consilience: The unity of knowledge. Vintage (1998).

[119] Mehra, K. If you claim India’s Covid death toll is 2x govt figure, it’s understandable. But not 10x. The Print*.* 2021 Apr 29.

[120] Saikia N, Kumar K, Das B (2023). Death registration coverage 2019–2021, India. Bull. World Health Organ..

[121] Thevar, S. Pune city sees fewer babies in 2020 than 2019. Hindustan Times. (2021).

[122] Thevar, S. Two years of Covid: How other diseases get back-burned. Hindustan Times. (2022).

[123] Bengrut, D. Fatal accidents on the rise in Pune; lifting of Covid curbs a factor. Hindustan Times. (2022).

[124] Sen, S. Fatalities on Mumbai roads plunge 36%, accidents 39% in 11 months of 2020. Times of India. 2021 Jan 14.

[125] Express News Service. Pune: Submit genuine documents for Covid death compensation or face legal action, warns PMC. The Indian Express. (2022).

[126] Surowiecki, J. The wisdom of crowds. Anchor (2004).

[127] Turiel J, Fernandez-Reyes D, Aste T (2021). Wisdom of crowds detects COVID-19 severity ahead of officially available data. Sci. Rep..

[128] Abrams, D. & Hogg, M. A. Social identifications: A social psychology of intergroup relations and group processes. Routledge (2006).

[129] Trotter, W. Instincts of the Herd in Peace and War. Fisher Unwin (1921).

[130] Dyer JR, Johansson A, Helbing D, Couzin ID, Krause J (2009). Leadership, consensus decision making and collective behaviour in humans. Philos. Trans. R. Soc. B Biol. Sci..

[131] Mackay, C. Extraordinary popular delusions and the madness of crowds (1841). Simon and Schuster (2012).

[132] Cinelli M, De Francisci MG, Galeazzi A, Quattrociocchi W, Starnini M (2021). The echo chamber effect on social media. Proc. Natl Acad. Sci..

[133] Terren L, Borge-Bravo R (2021). Echo chambers on social media: A systematic review of the literature. Rev. Commun. Res..

[134] Bavel JJ, Baicker K, Boggio PS, Capraro V, Cichocka A, Cikara M, Crockett MJ, Crum AJ, Douglas KM, Druckman JN, Drury J (2020). Using social and behavioural science to support COVID-19 pandemic response. Nat. Hum. Behav..

[135] van der Linden S (2022). Misinformation: Susceptibility, spread, and interventions to immunize the public. Nat. Med..

[136] Zarocostas J (2020). How to fight an infodemic. The Lancet..

[137] Zhang H, Maloney LT (2012). Ubiquitous log odds: a common representation of probability and frequency distortion in perception, action, and cognition. Front. Neurosci..

[138] Griffiths TL, Tenenbaum JB (2006). Optimal predictions in everyday cognition. Psychol. Sci..

[139] Huttenlocher J, Hedges LV, Duncan S (1991). Categories and particulars: prototype effects in estimating spatial location. Psychol. Rev..

[140] Lee MD, Danileiko I (2014). Using cognitive models to combine probability estimates. Judgm. Decis. Mak..

[141] Attneave F (1953). Psychological probability as a function of experienced frequency. J. Exp. Psychol..

[142] Chesney D, Bjalkebring P, Peters E (2015). How to estimate how well people estimate: Evaluating measures of individual differences in the approximate number system. Atten. Percept. Psychophys..

[143] Hvidberg, K. B., Kreiner, C. & Stantcheva, S. Social Positions and Fairness Views on Inequality. National Bureau of Economic Research (2020).

[144] Kruger J, Dunning D (1999). Unskilled and unaware of it: How difficulties in recognizing one's own incompetence lead to inflated self-assessments. J. Personal. Soc. Psychol..

[145] Landy D, Guay B, Marghetis T (2018). Bias and ignorance in demographic perception. Psychonom. Bull. Rev..

[146] Riederer, C., Hofman, J. M. & Goldstein, D. G. To put that in perspective: Generating analogies that make numbers easier to understand. In *Proceedings of the 2018 CHI Conference on Human Factors in Computing Systems 2018 Apr 21* (pp. 1–10).

[147] Schlichting, N., Damsma, A., Aksoy, E. E., Wächter, M., Asfour, T. & van Rijn, H. Temporal context influences the perceived duration of everyday actions: Assessing the ecological validity of lab-based timing phenomena. *J. Cogn.* **2**(1) (2018). 10.5334/joc.4 PMC6646943 31517220

[148] Sedlmeier P, Hertwig R, Gigerenzer G (1998). Are judgments of the positional frequencies of letters systematically biased due to availability?. J. Exp. Psychol. Learn. Mem. Cogn..

[149] Varey CA, Mellers BA, Birnbaum MH (1990). Judgments of proportions. J. Exp. Psychol. Hum. Percept. Perform..

[150] Becker J, Porter E, Centola D (2019). The wisdom of partisan crowds. Proc. Natl. Acad. Sci..

[151] Lorenz J, Rauhut H, Schweitzer F, Helbing D (2011). How social influence can undermine the wisdom of crowd effect. Proc. Natl. Acad. Sci..

[152] Henrich J, Heine SJ, Norenzayan A (2010). The weirdest people in the world?. Behav. Brain Sci..

[153] Taleb, N. N. The black swan: The impact of the highly improbable. Random house (2007).

[154] United Nations. The 17 Goals. Sustainable Development Goals. 10 August 2022. Accessed from: https://sdgs.un.org/goals

[155] Natarajan S, Subramanian P (2022). Systematic review of excess mortality in india during the Covid-19 pandemic with differentiation between model-based and data-based mortality estimates. Indian J. Commun. Med..

[156] Rukmini, S. Gauging pandemic mortality with civil registration data. The Hindu (2021).

[157] Khairnar, A. Cremation figure belies PMC count. Hindustan Times. (2021).

[158] Deshpande, M. & Hindocha, J. PMC, PCMC cremation count continues to belie administration’s death toll. Hindustan Times. (2021).

[159] Chaudhary, A. Gujarat, Telangana receive Covid death claims 9 & 7 times of official toll. The Times of India (2022).

[160] Khan, S. Gujarat Covid ex gratia claims cross 1 lakh, 87,000 approved. The Times of India. 2022 Feb 4.

