# Supplementary material for: Conventional and frugal methods of estimating COVID-19-related excess deaths and undercount factors
Source: Sci Rep. 2024 May 6;14:10378. doi: 10.1038/s41598-024-57634-6 (PMC11074152; doi:10.1038/s41598-024-57634-6)
Supplement: Supplementary file 1 — Supplementary Information. [file 41598_2024_57634_MOESM1_ESM.pdf]

Full title: Conventional and Frugal Methods of Estimating COVID-19-Related Excess Deaths and Undercount Factors

Authors: Abhishek M. Dedhe[1,2,3*], Aakash A. Chowkase[1,4], Niramay V. Gogate[1,5], Manas M. Kshirsagar[1,6], Rohan Naphade[1], Atharv Naphade[1], Pranav Kulkarni[1,7], Mrunmayi Naik[1], Aarya Dharm[1,8], Soham Raste[1], Shravan Patankar[1,9], Chinmay M. Jogdeo[1,10], Aalok Sathe[1,11], Soham Kulkarni[1,12], Vibha Bapat[1,13], Rohinee Joshi[1,14], Kshitij Deshmukh[1,15,16], Subhash Lele[1,17], Kody J. Manke-Miller[2], Jessica F. Cantlon[2,3], Pranav S. Pandit[1,18]

Affiliations:

[1]JPF Analytics, Jnana Prabodhini Foundation, Murrieta, California, United States of America

[2]Department of Psychology, Carnegie Mellon University, Pittsburgh, Pennsylvania, United States of America

[3]Center for the Neural Basis of Cognition, Carnegie Mellon University, Pittsburgh, Pennsylvania, United States of America

[4]Department of Psychology, University of California, Berkeley, California, United States of America

[5]Department of Physics and Astronomy, Texas Tech University, Lubbock, Texas, United States of America

[6]Institute of Clinical Neurobiology, University Hospital Würzburg, Würzburg, Germany

[7]Department of Electrical Engineering, California Institute of Technology, Pasadena, California, United States of America

[8]School of Computer Science and Engineering, University of Washington, Seattle, Washington, United States of America

[9]Department of Mathematics, University of Illinois, Chicago, Illinois, United States of America

[10]College of Pharmacy, University of Nebraska Medical Center, Omaha, Nebraska, United States of America

[11]Department of Brain and Cognitive Sciences, Massachusetts Institute of Technology, Cambridge, Massachusetts, United States of America

[12]Troy High School, Fullerton, California, United States of America

[13]Department of Biology, Indian Institute of Science Education and Research, Pune, Maharashtra, India

[14]Department of Mathematics, Indian Institute of Technology, Mumbai, Maharashtra, India

[15]Division of Molecular and Cellular Function, School of Biological Sciences, University of Manchester, Manchester, Greater Manchester, United Kingdom

[16]Department of Molecular Physiology and Biophysics, Pappajohn Biomedical Discovery Building (PBDB), University of Iowa, Iowa City, Iowa, United States of America

[17]Department of Mathematical and Statistical Sciences, University of Alberta, Edmonton, Alberta, Canada

[18]Department of Population Health and Reproduction, School of Veterinary Medicine, University of California, Davis, Davis, California, United States of America

*Corresponding authors: Abhishek M. Dedhe and Pranav S. Pandit

Emails: adedhe@andrew.cmu.edu, pspandit@ucdavis.edu, analytics@jnanaprabodhinifoundation.org

Supporting Information

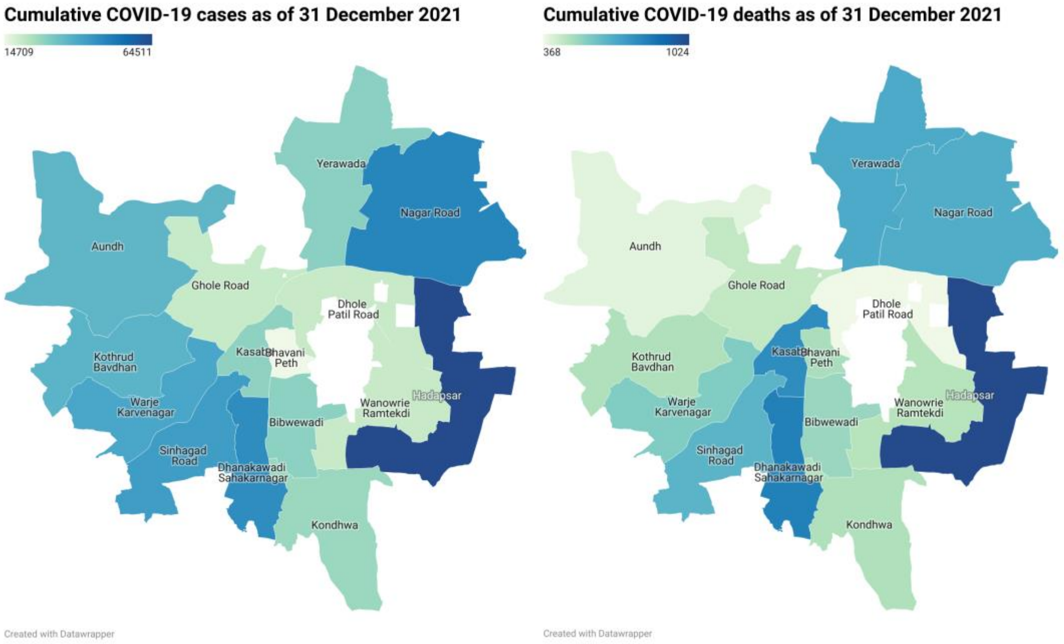

Figure S1. Officially reported COVID-19 cases and mortality in each ward of Pune. Left: Cumulative number of COVID-19 cases reported in Pune from March 2020 through December 2021. Right: Cumulative number of COVID-19 deaths reported in Pune from March 2020 through December 2021. Note that "reported cases" refers to "incidence".

Clarification about accuracy of estimates

We used multiple methods to compute COVID-19-related excess death estimates and undercount factors for Pune. These methods point to similar conclusions and therefore demonstrate "convergence", "methodological triangulation" (117), or "consilience" (118). Thus, even though each method may not be highly reliable when considered individually, the overlap from these unrelated methods points to the strength and trustworthiness of the conclusion.

Clarification about precision of estimated undercount factors

We computed an aggregate undercount factor ($\mu = 1.6$; 95% CI: 1.1 – 2.5) that is sufficiently precise or "narrow" enough to provide real-world insights. Undercount factors are typically used to evaluate the performance of public health data recording systems during disease outbreaks or natural disasters. Pune's performance is comparable to some of the world's best healthcare systems (including countries in the Global North), that saw undercount factors around 1.5 – 2 during the COVID-19 pandemic (2, 8, 113; **Fig. S2**). More specifically, our 95% confidence interval implies that Pune's undercount factor could be as low as 1.1, suggesting near-perfect data recording. Given that Maharashtra has a robust data infrastructure with an estimated 100% pre-pandemic death registration coverage (18, 120), it is plausible that Pune's public health system was resilient enough to avoid undercounting deaths, especially during "non-wave" periods of the COVID-19 pandemic. However, during times of extreme stress and pressure such as the deadly second wave of April-May 2021 (the "Delta wave"), Pune's performance may have deteriorated. Discrepancies between reported COVID-19 cremations and reported COVID-19 deaths suggest that Pune's undercount factor may have been as high as 2.2 during the Delta wave (**Table S3**). This value lies near the upper end of our 95% confidence interval, thus providing an additional real-world "sanity check" about the precision of our computed margin of error. Our 95% confidence interval spanned 1.1 through 2.5, i.e., a range of 1.4. Compared to over 400 locations across the world studied in Wang et al., 2022 (8), Pune's 95% confidence interval range lies in the bottom third (**Fig. S3**). Pune ranks 289th out of 441 countries and regions. It ranks exactly one spot above Delhi, twelve spots above Maharashtra state, and three spots below India and South-East Asia.

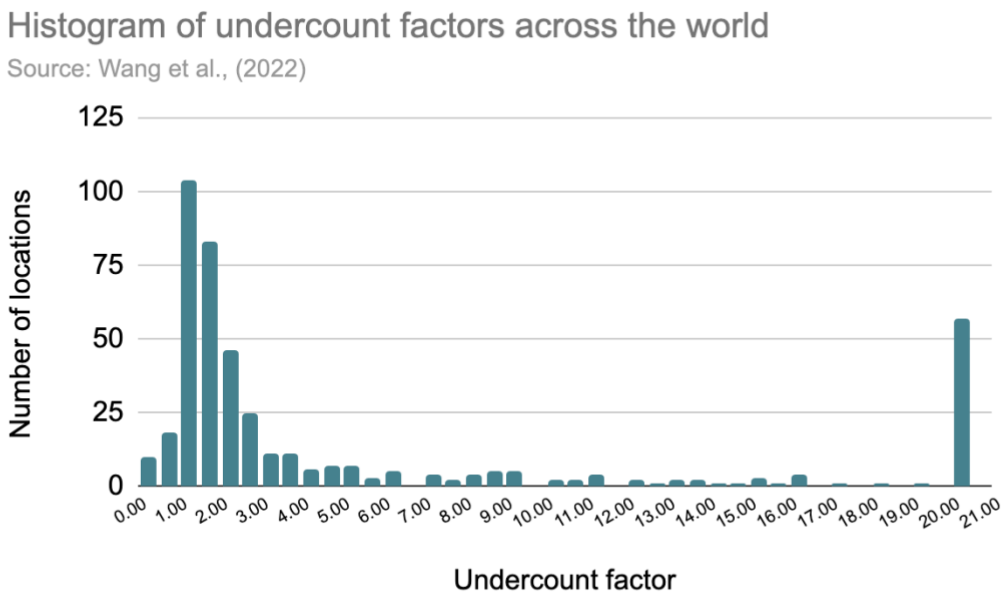

Figure S2. Global undercount factors from Wang et al., 2022 (8). All values equal to and greater than 20 were grouped together in the same bucket. Pune = 1.6.

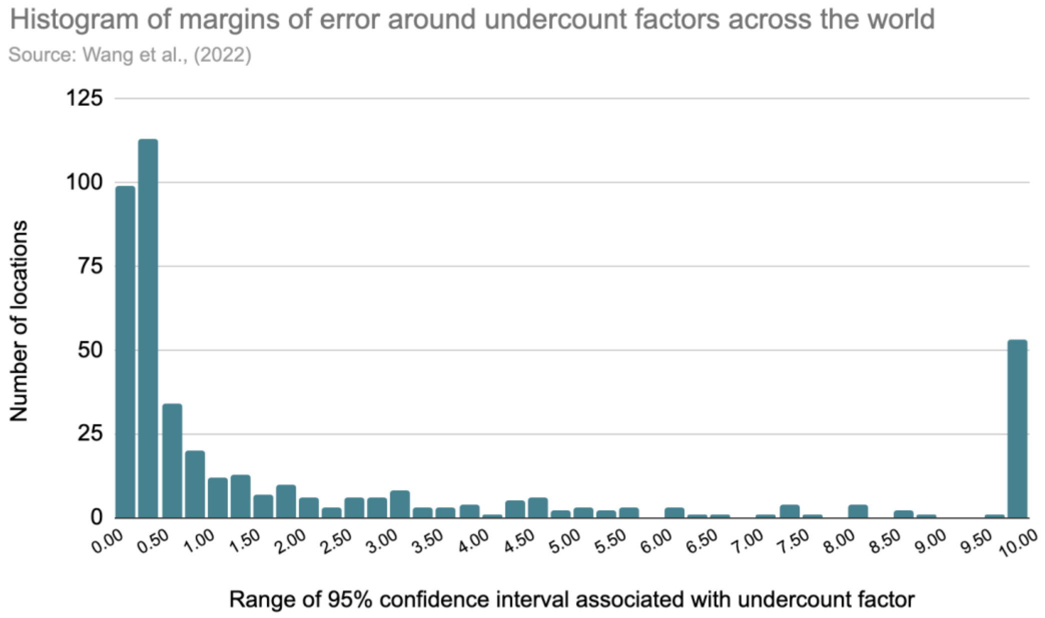

Figure S3. Ranges of 95% confidence intervals associated with undercount factors from Wang et al., 2022 (8). All values equal to and greater than 10 were grouped together in the same bucket. The width of the CI based on our aggregate estimate for Pune was 1.4.

Statistical models: details and assumptions

We ran simple time series analyses such as ARIMA before implementing the statistical and epidemiological models (see attached .R code and .html file for more details).

Farrington surveillance algorithm

The model was implemented using the *surveillance* package in R (105,111). Specifically, we used the `algo.farrington` function. We set the `control` arguments (i.e., the model parameters) as `b=5`; `w=1`. Here, `b` refers to the number of years back in time to include when computing the number of expected deaths, and `w` refers to the window size, i.e., the number of months to include before and after the current month. We chose `b=5` because we possessed data from 6 pre-pandemic years (2014, 2015, 2016, 2017, 2018, and 2019). Note that `b=5` not `b=6` even though 6 pre-pandemic years are considered. This is because the code begins indexing from 0. We chose `w=1` because we wished to maximize the granularity of the data we possessed, especially because we only possessed monthly data and not weekly data. Therefore, we chose the smallest window possible.

Overdispersed Poisson model

The model was implemented using the *excessmort* package in R (104). Specifically, we used the `computed_expected` and the `excess_model` functions. We set the arguments (i.e., the model parameters) as `harmonics=1` in `computed_expected` and `knots.per.year=2` in `excess_model`. Here, `harmonics` refer to the number of harmonics used to model a seasonal trend accounting for a seasonal variation in deaths, and `knots.per.year` refer to the amount of smoothing in the cubic spline used to model the gradual trend accounting for the increasing life

expectancy. Sinusoidal curves are modeled with `harmonics=1` whereas more complex curves are modeled with more harmonics (e.g., `harmonics=2`). We chose `harmonics=1` in our model because we preferred model simplicity; this came at the cost of not being able to model more complex patterns. `Harmonics=1` corresponds to the lowest possible integer value that the parameter could take. Large `knots.per.year` values (~12 per year) provide enough flexibility to detect both natural disasters and outbreaks. Smaller `knots.per.year` values (~6 per year) fit smoother splines, with more power to detect subtle indirect effects – they have lower false discovery rates. We chose `knots.per.year =2` because we wanted lower false discovery rates; these came at the cost of lower model flexibility.

Assumption of constant pre-pandemic number of deaths

Two of the statistical models we used: a) the simple averaging technique and b) the Farrington surveillance algorithm assumed that pre-pandemic number of deaths was effectively constant over time. We assessed this assumption by running a simple linear regression using the `lm()` function in `R`. We found a weak (R^2 = .13) yet significant (p = .002) increase in the overall number of observed deaths from 2014 through 2019 (**Fig. S4**). Model results:

X predicted Y, R^2 = .13, $F(1,70)$ = 10.59, p = .002.
β = 6.29, p = .002, α = 2249.74, p < .001.

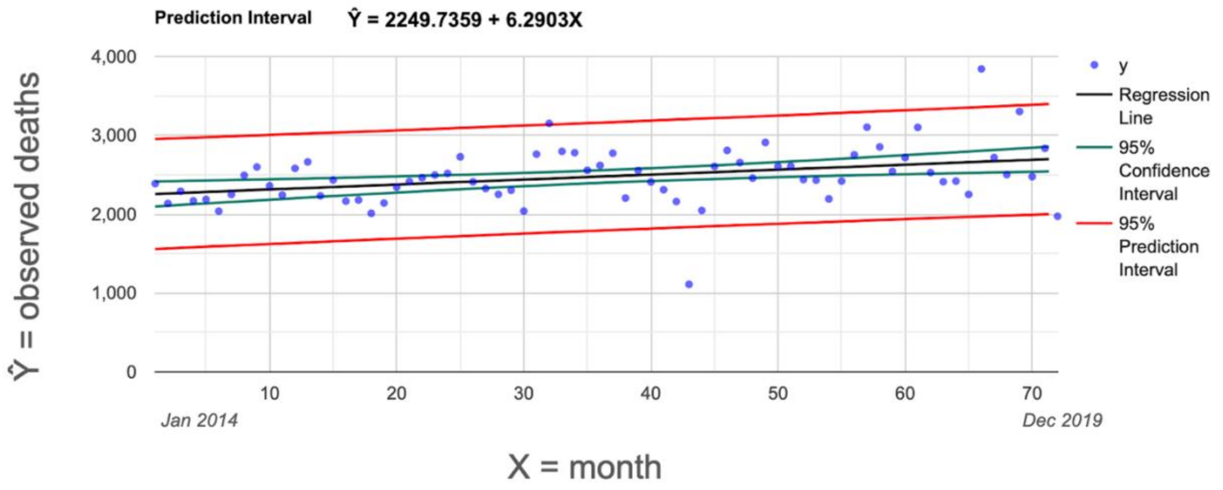

Figure S4. Monthly observed all-cause deaths in the Pune Municipal Corporation (PMC) during pre-pandemic times from January 2014 through December 2019. Indexing of months begins at 1. The 95% confidence interval and the 95% prediction interval are based on the fitted line.

Thus, the model assumption was mildly violated. However, both models showed relatively robust performance despite this violated assumption. We assessed model performance by computing the predicted (i.e., expected) number of deaths along with 95% prediction intervals. For the simple average model, the 95% prediction interval was defined as $[\mu \pm Z\sigma]$ where σ is the standard deviation around the predicted estimate and $Z = 1.96$ (i.e., the 97.5th percentile of a standard normal distribution). For the Farrington surveillance algorithm, the lower bound for the margin of error was the one-sided 95% prediction interval and the upper bound was computed using average expected deaths. As described in the main text, predictions for pandemic years were based on trends from all pre-pandemic years, i.e., 2014 through 2019. We assessed whether model performance was robust to the violated assumption by testing whether the number of observed deaths for *pandemic* years was *significantly higher* than the number of predicted (i.e., expected) deaths for that time period. Additionally, we assessed whether the number of observed deaths for

pre-pandemic years was *not significantly higher* than the number of predicted (i.e., expected) deaths for that time period. Thus, robust model performance would be characterized by:

a) Successful prediction of excess deaths during pandemic years
b) No prediction of excess deaths during pre-pandemic years

Note that we normalized the predicted (i.e., expected) deaths, the lower and upper bounds of the computed 95% prediction intervals, and the observed deaths by dividing these values by the number of predicted (i.e., expected) deaths. We found that performance of both the simple average model (**Fig. S5**) and the Farrington surveillance algorithm (**Fig. S6**) depended upon the amount of data used. When we used data from six years (2014-2019) or five years (2015-2019), both models showed observed deaths outside the bounds of the 95% prediction interval and thus successfully predicted excess deaths during the pandemic. For most data subsets that were smaller such as four years (2016-2019), three years (2017-2019), or two years (2018-2019), both models did not show observed deaths outside the bounds of the 95% prediction interval. In summary, model performance is relatively robust even when the underlying assumption of effectively constant pre-pandemic number of deaths is violated. However, model performance is sensitive to the amount of underlying data – requiring at least four years of data to robustly withstand model violations. In addition to assessing whether there was robust model performance characterized by successful prediction of excess deaths during pandemic years, we also tested whether the models did not predict any excess deaths during pre-pandemic years. We found that the performance of both the simple average model (**Fig. S7**) and the Farrington surveillance algorithm (**Fig. S8**) depended upon the amount of data used. When we used data from five years (2014-2018) or four years (2014-2017), both models showed observed deaths within the bounds of the 95% prediction interval and thus did not predict any excess deaths during various pre-pandemic years. For most data subsets

that were smaller such as three years (2014-2016), or two years (2014-2015), both models sometimes showed observed deaths outside the bounds of the 95% prediction interval. In summary, model performance is relatively robust even when the underlying assumption of effectively constant pre-pandemic number of deaths is violated. Similar to **Fig. S5** and **Fig. S6**, model performance is sensitive to the amount of underlying data – requiring at least four years of data to robustly withstand model assumption violations.

In summary, both models assume constant pre-pandemic number of deaths because their data requirement, unlike the overdispersed Poisson model's, does not include underlying population data. Incorporating these data shows that pre-pandemic death rates were effectively constant over time (**Fig. S9**). Even though the simple average model does not require or use underlying population data, these data may be incorporated into the model predictions for more robust results, especially when such data are readily available.

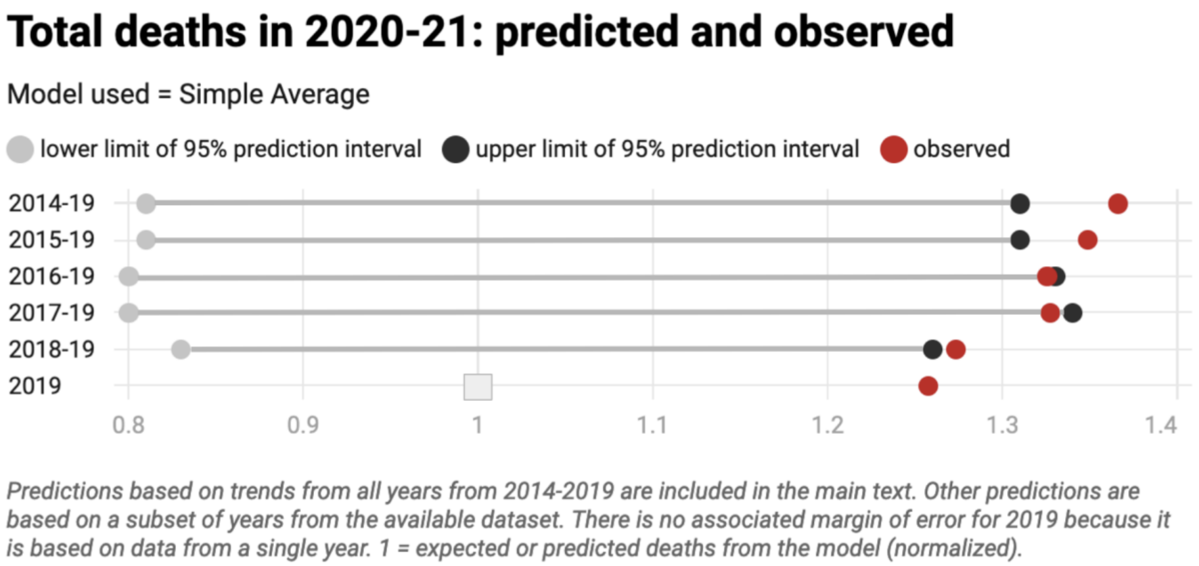

Figure S5. Assessing robustness to violated model assumption in the simple average model during pandemic years.

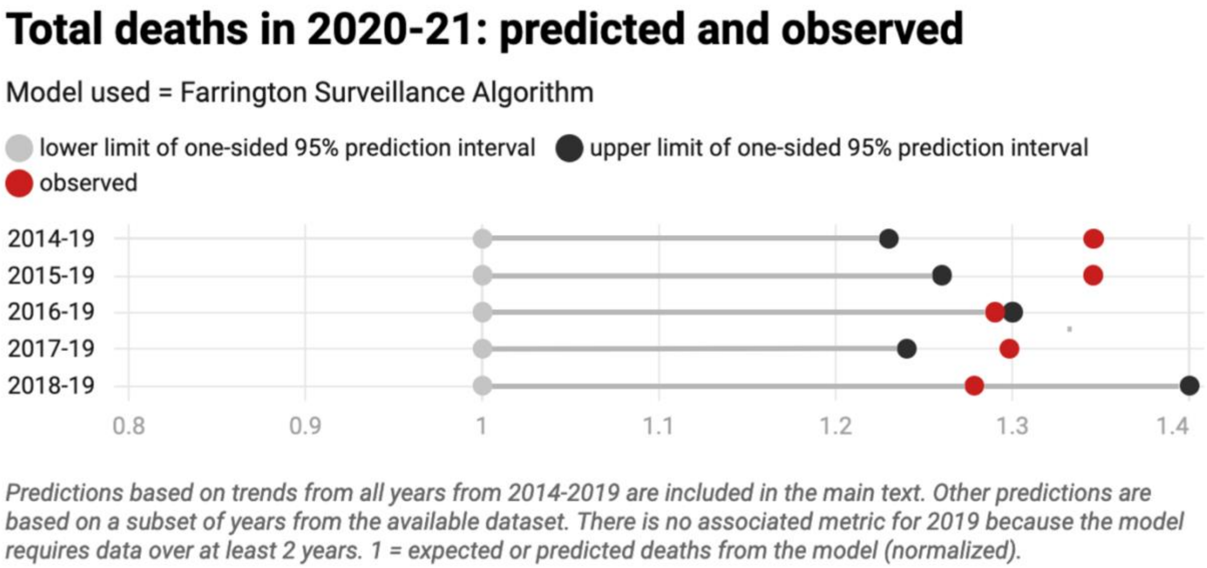

Figure S6. Assessing robustness to violated model assumption in the Farrington surveillance algorithm during pandemic years.

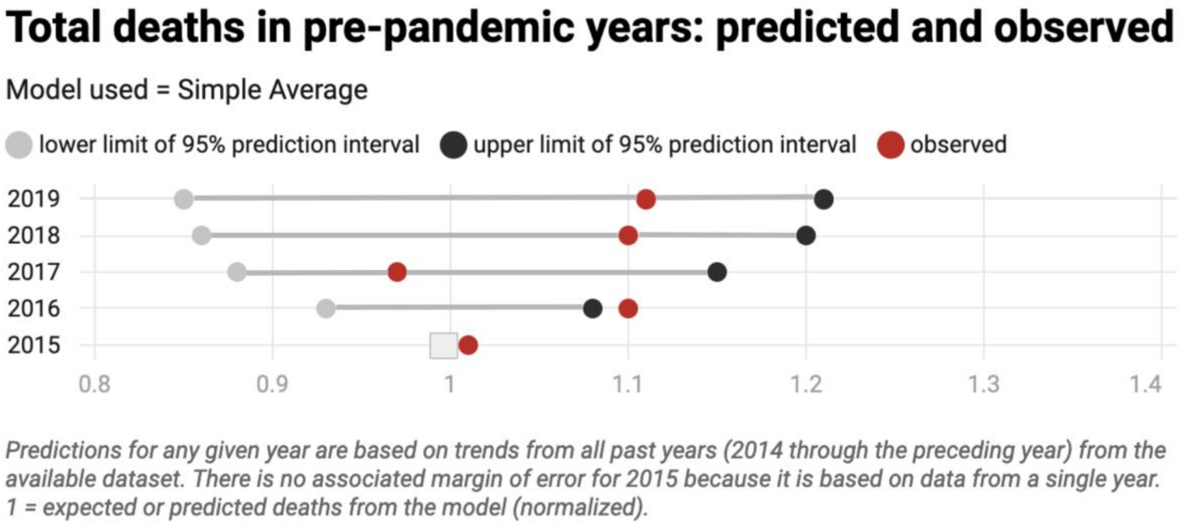

Figure S7. Assessing robustness to violated model assumption in the simple average model during pre-pandemic years.

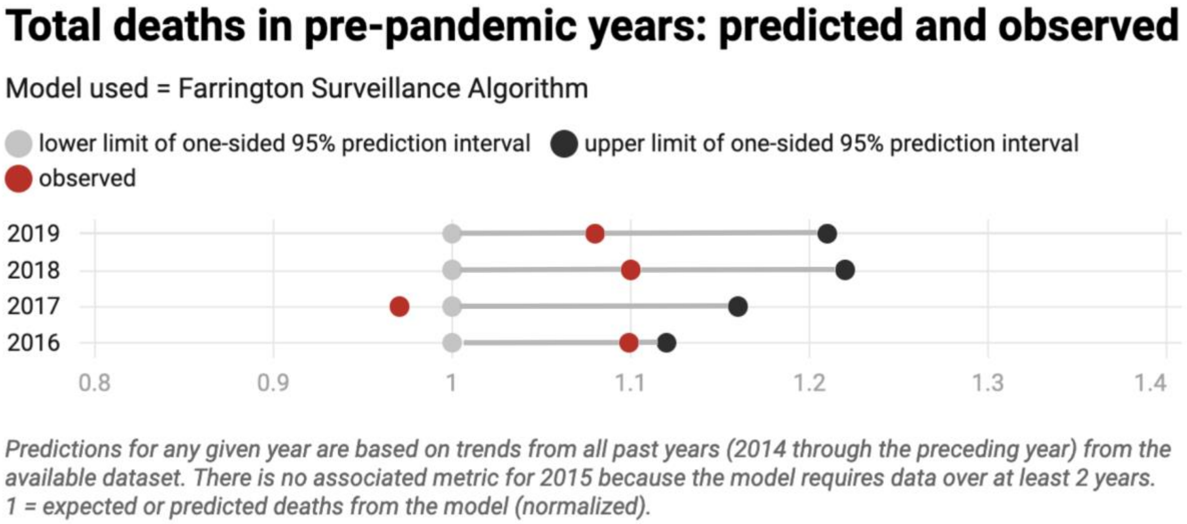

Figure S8. Assessing robustness to violated model assumption in the Farrington surveillance algorithm during pre-pandemic years.

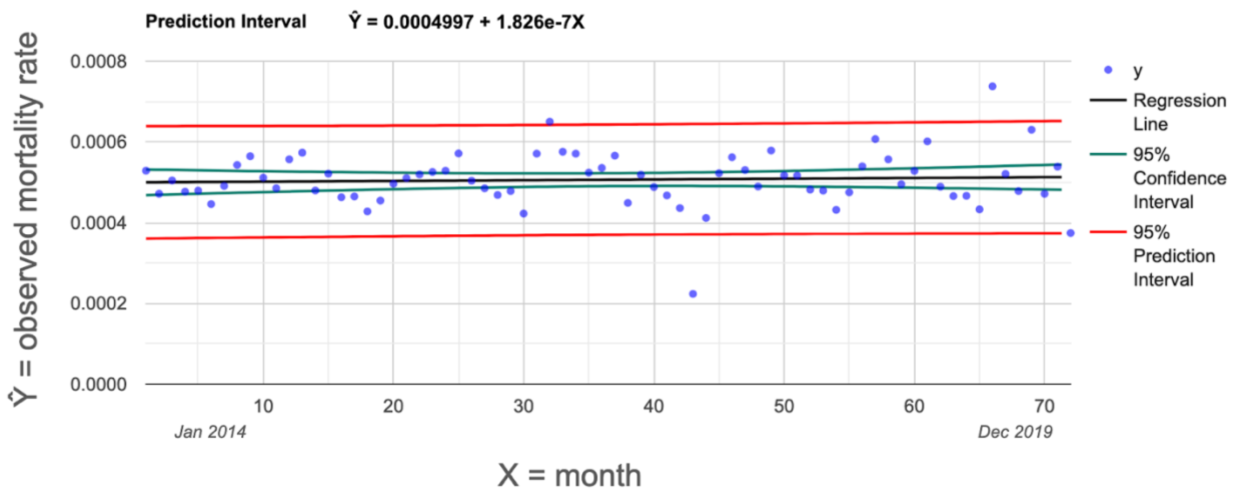

Figure S9. Monthly observed mortality rate in the Pune Municipal Corporation (PMC) during pre-pandemic times from January 2014 through December 2019. Indexing of months begins at 1. The 95% confidence interval and the 95% prediction interval are based on the fitted line.

Model results:

X predicted Y, R^2 = .0032, $F(1,70)$ = 0.22, p = .637.
β = .8e-7, p = .637, α = 0.0005, p < .001.

Death compensation claims: details and assumptions

Because there isn't a well-justified statistical approach to compute a margin of error for this method, we heuristically estimated a range of plausible undercount factors. We focused on the following five locales (**Fig. S10**):

1. Pune city (1.4 – 1.7): The undercount factor used in the main text was computed as the ratio of reported death compensation claims to reported COVID-19 deaths in Pune as of January 2022 (113). This value was considered to be the lower limit of the plausible range of undercount factors for Pune. The upper limit was similarly computed using reported values for Pune from April 2022 (125).
2. Cities in Maharashtra (0.9 – 2.1): As described in the main text, the undercount factor was computed based on death compensation claims from 7 cities in Maharashtra state (excluding Pune) as of January 2022 (113). We calculated the standard error for the undercount factors, thus generating a range of plausible undercount factors for cities in Maharashtra. This standard error was used to compute a 95% confidence interval for Pune. The lower and upper bound of this confidence interval was considered to be the plausible range of undercount factors for Pune.
3. Maharashtra state (1.51 – 1.59): The undercount factor was computed as the ratio of reported death compensation claims to reported COVID-19 deaths in Maharashtra state as of January 2022 (159). This value was considered to be the lower limit of the plausible range of undercount factors for Pune. The upper limit was similarly computed using reported values for Maharashtra state from February 2022 (160).
4. Indian cities (1.6 – 5.7): Other sources (10,156) used statistical modeling with pre-pandemic all-cause mortality data to compute undercount factors for various major Indian

cities (**Table S1**). These undercount factors were not based on death compensation claims. We calculated the standard error for these undercount factors, thus generating a range of plausible undercount factors for cities of India. This standard error was used to compute a 95% confidence interval for Indian cities. The lower and upper bound of this confidence interval was considered to be the plausible range of undercount factors for Pune.

5. Indian states (0.7 – 5.2): Undercount factors were computed as the ratio of reported death compensation claims to reported COVID-19 deaths in 8 Indian states (excluding Maharashtra) as of January 2022 (159). We calculated the standard error for the undercount factors, thus generating a range of plausible undercount factors for states of India. This standard error was used to compute a 95% confidence interval for Indian states. The lower and upper bound of this confidence interval was considered to be the plausible range of undercount factors for Pune.

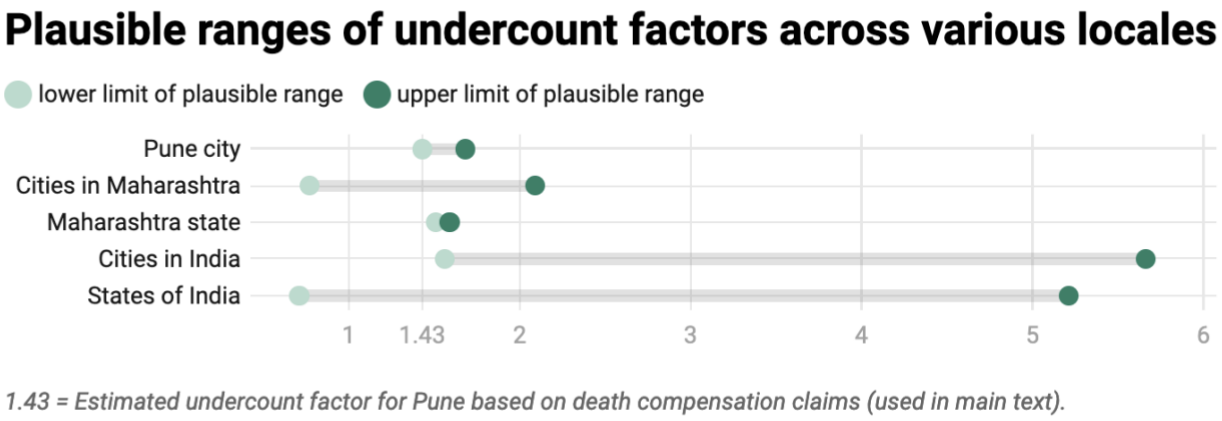

Figure S10. Heuristically estimated plausible ranges of undercount factors for Pune.

City	Undercount factor	Wave	Margin of Error
Ahmedabad	6.1	1	1.9 to 10.3
Bangalore	1.2	1	2.7 to 3.2
Bangalore	2.9	2	0.8 to 1.6
Chennai	1.9	1	1.8 to 2.0
Chennai	2.6	2	2.0 to 2.6
Delhi	1	1	*NA*
Delhi	3.4	2	*NA*
Hyderabad	10	1, 2	9.6 to 10.4
Kolkata	1.1	1	1.07 to 1.13
Kolkata	4.7	2	2.7 to 6.7
Mumbai	1.2	1	1.1 to 1.3
Mumbai	2.6	2	2.0 to 3.2
Nagpur	1.5	1	1.0 to 2.0

Table S1. Undercount factors for some major Indian cities (10,156).

Wisdom of crowds survey: details

	Males	151 (54%)
**Gender**	Females	129 (46%)
	18-35	115 (41%)
**Age**	36-55	115 (41%)
	55+	50 (18%)
	Marathi	139 (50%)
**Survey language**	English	141 (50%)
	1-2	16 (6%)
**Number of rooms in home**	3	60 (22%)
	4	102 (36%)
	4+	102 (36%)
	Students	71 (25%)
	Currently employed	175 (63%)
**Occupation**	Healthcare workers	25 (9%)
**Current or past COVID-19 +ve**		101 (36%)
**Total sample size**		**280**

Table S2. Demographic details of wisdom of crowds public survey respondents.

.

Aggregate estimate using bootstrapping: details

The histogram below shows the mean undercount factor computed from the simple bootstrap where we ran 10,000 iterations of random sampling with replacement. We sampled from normal distributions with these parameters: a) simple average — $\mu = 2.25$, $\sigma = 0.65$; b) Farrington surveillance — $\mu = 1.01$, $\sigma = 0.6$; c) overdispersed Poisson — $\mu = 1.67$, $\sigma = 0.65$; and d) death compensation claims — $\mu = 1.43$, $\sigma = 0.9$. We assumed no correlation between the different statistical and epidemiological models.

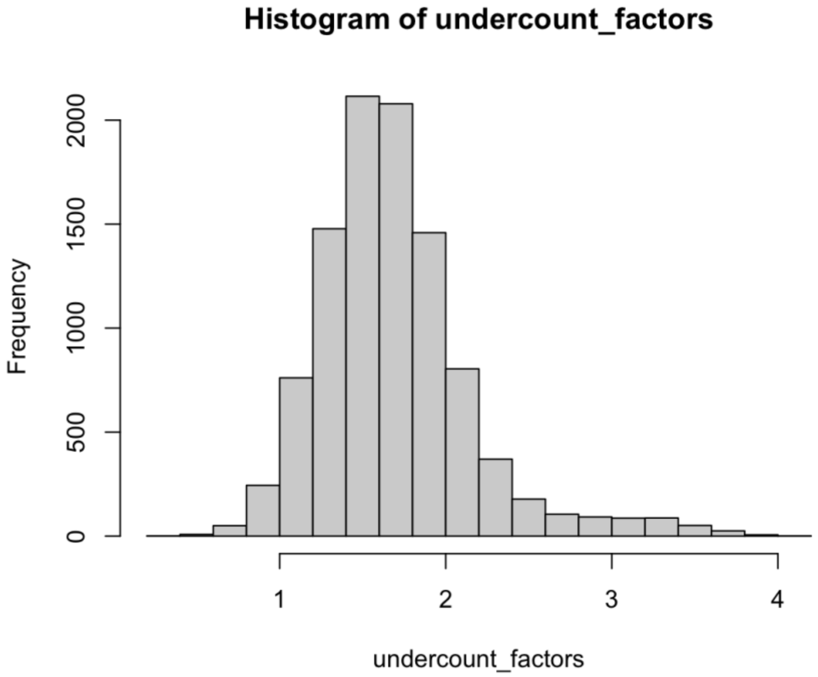

Figure S11. Results of the simple bootstrap (10,000 iterations of random sampling with replacement).

Estimating excess mortality using COVID-19 cremation counts obtained from media reports

Discrepancies between cremation count and officially reported deaths, and estimates of undercount factors during the second wave of the COVID-19 pandemic in Pune and its sister city, the PCMC (Pimpri Chinchwad Municipal Corporation). Some of the estimates were approximate (149,150).

**Date or timespan**	**Excess COVID-19-related deaths**		**Undercount factor**
	**Official COVID-19 Deaths**	**COVID-19 Cremation Count**	**Estimate**
April 17, 2021	53	>100	1.9
April 18, 2021	54	>100	1.9
April 24, 2021	55	~120	2.2
April 25, 2021	55	~120	2.2
April 1 through April 24, 2021 (PCMC)	622	1,820	2.9
**Aggregate estimate**	*NA*	*NA*	**2.2**

Table S3. Discrepancies between observed COVID-19 cremation counts and officially reported COVID-19 deaths, and estimates of undercount factors during the COVID-19 pandemic in Pune during April 2021. The number of COVID-19 cremation counts observed in Pune based on the media report was approximate (157,158).

Proxy factor	2019	2020	Dip
AIDS blood tests	~100,000	68,000	32%
Tuberculosis cases	~7,000	4,900	30%
Road accidents	791	482	39%
Births	52,669	50,544	4%

Table S4. Proxy estimates of fluctuations in death registration coverage in Pune from media reports (121-123).
